# Phosphorylation of XPD drives its mitotic role independently of its DNA repair and transcription functions

**DOI:** 10.1126/sciadv.abp9457

**Published:** 2022-08-17

**Authors:** Emmanuel Compe, Evanthia Pangou, Nicolas Le May, Clémence Elly, Cathy Braun, Ji-Hyun Hwang, Frédéric Coin, Izabela Sumara, Kwang-Wook Choi, Jean-Marc Egly

**Affiliations:** ^1^ Institut de Génétique et de Biologie Moléculaire et Cellulaire (IGBMC), Expression et Réparation du Génome, Equipe labellisée Ligue contre le Cancer, CNRS/INSERM/Université de Strasbourg, BP 163, Illkirch Cedex, C. U., 67404 Strasbourg, France.; ^2^ Institut de Génétique et de Biologie Moléculaire et Cellulaire (IGBMC), Cycle Cellulaire et Signalisation de l’Ubiquitine, CNRS/INSERM/Université de Strasbourg, BP 163, Illkirch Cedex, C. U., 67404, Strasbourg, France.; ^3^ Department of Biological Sciences, Korea Advanced Institute of Science and Technology, Daejeon, Korea.; ^4^ College of Medicine, National Taiwan Institute, Taipei 10051, Taiwan.

## Abstract

The helicase XPD is known as a key subunit of the DNA repair/transcription factor TFIIH. However, here, we report that XPD, independently to other TFIIH subunits, can localize with the motor kinesin Eg5 to mitotic spindles and the midbodies of human cells. The XPD/Eg5 partnership is promoted upon phosphorylation of Eg5/T926 by the kinase CDK1, and conversely, it is reduced once Eg5/S1033 is phosphorylated by NEK6, a mitotic kinase that also targets XPD at T425. The phosphorylation of XPD does not affect its DNA repair and transcription functions, but it is required for Eg5 localization, checkpoint activation, and chromosome segregation in mitosis. In XPD-mutated cells derived from a patient with xeroderma pigmentosum, the phosphomimetic form XPD/T425D or even the nonphosphorylatable form Eg5/S1033A specifically restores mitotic chromosome segregation errors. These results thus highlight the phospho-dependent mitotic function of XPD and reveal how mitotic defects might contribute to XPD-related disorders.

## Teaser

This work describes how the DNA repair/transcription factor XPD also intervenes in mitosis.

## INTRODUCTION

The human xeroderma pigmentosum group D gene *XPD* (also named *ERCC2*) encodes an adenosine triphosphate (ATP)–dependent 5′-3′ helicase of 760 amino acids (*1*). This protein is known to be one of the 10 subunits of the TFIIH (Transcription Factor II H) complex, which is involved in nucleotide excision repair (NER) pathway and in transcription mediated by the RNA polymerase II (RNAPII) (*2*). In NER, besides its contribution to reveal ultraviolet (UV)–induced DNA damage, the helicase activity of XPD opens the double-stranded DNA to further allow the removal of the damaged oligonucleotide by XPG and XPF (Xeroderma Pigmentosum G and F) endonucleases (*1*, *3*). In transcription, while its helicase activity is dispensable, XPD has a structural function by bridging the cyclin-dependent kinase (CDK)–activating kinase (CAK) subcomplex [containing the cyclin H, MAT (Ménage à trois 1), and the kinase CDK7] to the core of TFIIH (XPB, p62, p52, p44, p34, and p8/TTDA) through an interaction with MAT1 and p44, respectively.

In addition, XPD can be found within TFIIH-independent complexes. In particular, XPD can be associated to the CAK without the presence of the core-TFIIH (*4*–*6*). This XPD/CAK association inhibits CAK activity (*7*), which is required during cell cycle by phosphorylating CDKs (including CDK1, CDK2, CDK4, and CDK6). In *Drosophila*, the Xpd inhibitory action on CAK activity seems to be circumvented by the association of Xpd to Mms19 (*8*). XPD and MMS19 were found in human cells associated with the adenine nucleotide translocase ANT2 and the cytosolic iron-sulfur protein assembly (CIA) machinery factors CIAO1 and macrophage inflammatory protein 18 in a complex named MMS19-MIP18-XPD (MMXD), which contributes to proper chromosome segregation in mitosis (*9*). Together, these observations suggest that XPD might play cellular functions independently of its presence within TFIIH. However, it remains unclear how XPD itself contributes to cell division and which molecular mechanisms drive the switch, allowing XPD to participate to these distinct cellular processes.

Mutations in the *XPD* gene result in different human autosomal recessive disorders (online Mendelian Inheritance in Man number: 126340), such as XP and trichothiodystrophy (TTD). Patients with XP-D develop severe phenotypes including neurological abnormalities and numerous skin defects ranging from excessive freckling to multiple skin cancers (*10*). These patients can sometimes develop XP combined with Cockayne syndrome, which associates XP phenotypes with severe dwarfism, mental retardation, and skeletal abnormalities (*11*). The principal hallmark of TTD is dry sparse hairs and brittle nails, but these patients can also develop other symptoms including ichthyosis, intellectual disability, reduced stature, and hypogonadism (*12*, *13*). Numerous studies were undertaken to determine the molecular and phenotypic consequences of XPD mutations. Although, until now, diseases resulting from XPD mutations are considered to be essentially related to DNA repair disorders (*14*), the various XPD functions suggest that defects in other cellular processes may contribute the pathophysiological process.

Here, we show that during mitosis, XPD localizes differently to other TFIIH subunits and that it interacts with Eg5, a motor kinesin protein required for establishing bipolar spindle (*15*, *16*). This XPD/Eg5 partnership is promoted upon phosphorylation of Eg5/T926 by the major mitotic kinase CDK1, and conversely, it is reduced once Eg5/S1033 is phosphorylated by NEK6 (never in mitosis gene A-related kinase 6). In addition to Eg5, we show that NEK6 phosphorylates XPD at its threonine-425 (T425) residue, which promotes the association of XPD with the CAK module of TFIIH, revealing a fine-tuned regulatory process that conditions the partnerships of XPD and in extenso its role in mitosis. Notably, XP-D patient cells bearing mutations affecting the interaction between XPD and Eg5 display defective mitotic progression including chromosome segregation errors, which partially result from deficient spindle assembly checkpoint (SAC) activation. These mitotic defects are rescued upon overexpression of the phosphomimetic form XPD/T425D and of the nonphosphorylatable form Eg5/S1033A. Together, these results highlight a TFIIH-independent mitotic function for XPD that is disrupted when XPD is mutated, suggesting that in addition to DNA repair and transcription defects, mitotic deficiencies contribute to XP-D phenotypes.

## RESULTS

### XPD colocalizes and interacts with Eg5 during mitosis

**Fig. 1.**
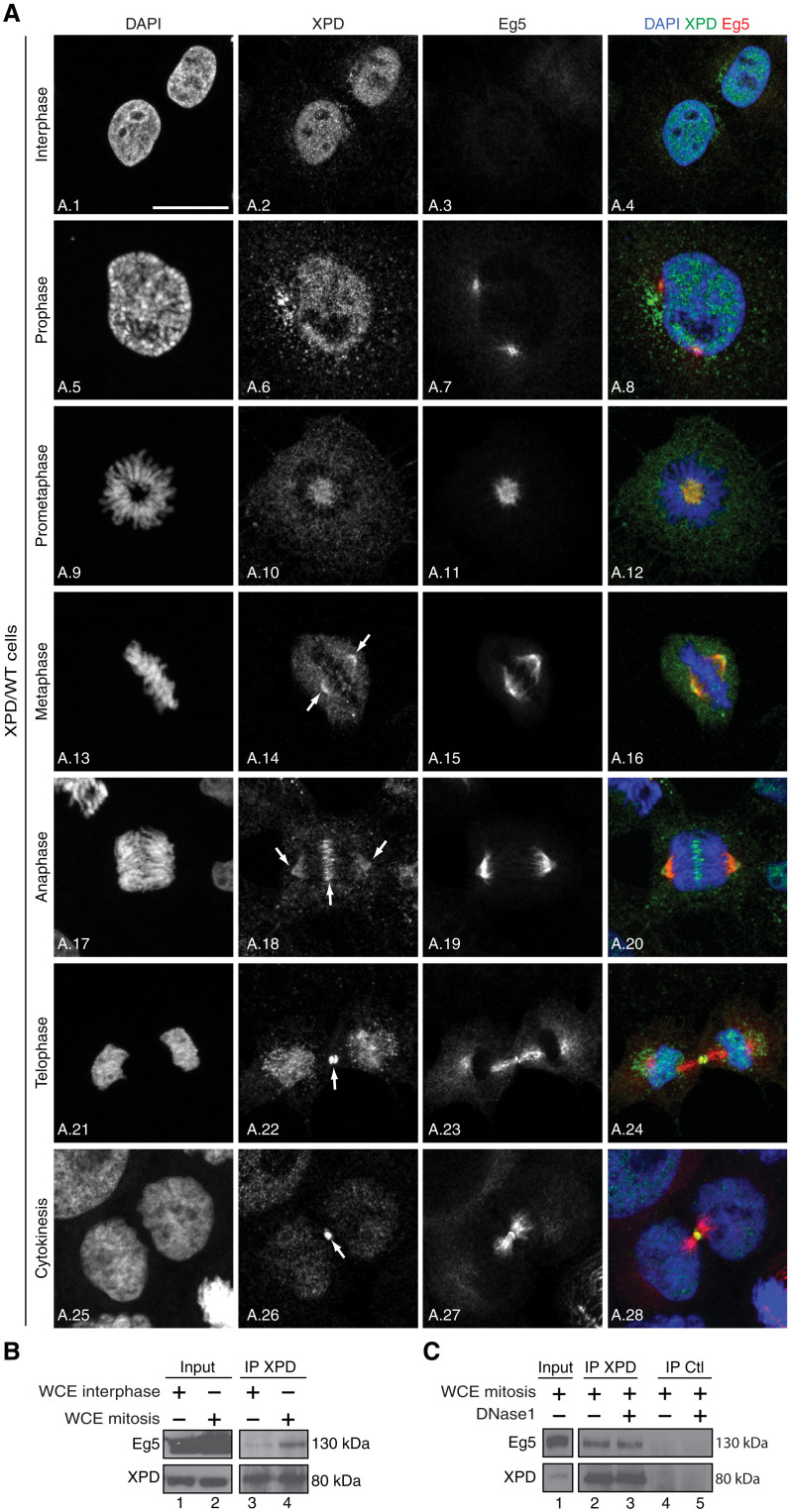
XPD colocalizes and interacts with Eg5. (**A**) Immunofluorescence analysis of XPD and Eg5 during interphase and different mitotic phases. Human XPD/WT cells were synchronized by double thymidine block and release, collected 9 hours after release, and analyzed by confocal microscopy at interphase, prophase, prometaphase, metaphase, anaphase, and telophase/cytokinesis. The arrows point to the localization of XPD at the mitotic spindle, the midzone, and at the midbody. Scale bar, 5 μm. (**B**) Whole-cell extracts (WCEs) were isolated from XPD/WT cells in interphase and mitosis (cells were treated 16 hours with nocodazole and collected 90 min after nocodazole release). After immunoprecipitation with anti-XPD (IP XPD), the coimmunoprecipitated proteins were resolved by SDS–polyacrylamide gel electrophoresis (SDS-PAGE) and blotted with anti-XPD and anti-Eg5. The results are representative of two independent experiments. (**C**) Whole-cell extracts were isolated from XPD/WT cells in mitosis [as indicated (B)], treated (when indicated, +) with deoxyribonuclease 1 (DNase1) (5 μg), and incubated (16 hours, 4°C) with anti-XPD (IP XPD) or irrelevant immunoglobulin G (IgG; IP Ctl) bound to magnetic beads. After washes, the coimmunoprecipitated proteins were resolved by SDS-PAGE and blotted with anti-XPD and Eg5. The results are representative of two independent experiments.

To gain first insights into the role of XPD during mitosis, we first analyzed its localization throughout the cell cycle. Confocal microscopy analysis of XPD/wild-type (WT) (HeLa) cells (Fig. 1A) showed that while XPD was mostly nuclear during interphase (images A.1 to A.2; as a factor involved in transcription and DNA repair) (*2*), its localization was dynamically changing during mitotic progression. XPD was essentially nuclear in prophase (images A.5 to A.6), but during prometaphase (images A.9 to A.10) and metaphase (images A.13 to A.14), XPD was excluded from the chromosomes with a substantial fraction being enriched at the mitotic spindle. While its localization persisted at the mitotic spindle, XPD also localized at the midzone in anaphase (images A.17 to A.18). XPD enriched at the midbody in telophase (images A.21 to A.22) contrary to its partner p44 (the regulatory subunit of the XPD helicase within TFIIH) and the CAK module (which phosphorylates CDKs, as well as RNAPII and nuclear receptors; fig. S1A, images A.1 to A.4, and A.5 to A.8) (*2*). Similarly, MMS19 (a partner of XPD within the mitotic MMXD complex having partial localization at the mitotic spindle in metaphase; fig. S1B) (*9*) did not localize at the midbody (fig. S1A, images A.9 to A.12), highlighting the fact that XPD might be found in mitosis independently from the CAK, TFIIH, and MMXD. However, we were surprised to find that XPD partially colocalized with the key mitotic motor kinesin Eg5 at microtubules in prometaphase (Fig. 1A, images A.9 to A.12), metaphase (images A.13 to A.16), and anaphase (images A.17 to A.20) and at the midbody in telophase (images A.21 to A.28). This colocalization of XPD and Eg5 during mitosis prompted us to investigate possible partnership between them. Immunoprecipitation assays from whole-cell extracts of XPD/WT cells synchronized in mitosis revealed that endogenous XPD interacted with Eg5 (Fig. 1B, lane 4); note that a truncated form of human recombinant XPD (XPD 444-760) coimmunoprecipitated with Eg5 (fig. S1C, lane 5), suggesting that at least the C-terminal part of XPD might interact with Eg5. In addition, we observed that deoxyribonuclease 1 treatment of whole-cell extracts from XPD/WT cells in mitosis did not affect the XPD/Eg5 partnership (Fig. 1C, lane 3), and the presence of single-stranded DNA, which is known to tightly bind XPD (*17*, *18*), did not influence the binding of recombinant XPD with Eg5 (fig. S1D, lane 5).

### Phosphorylation of Eg5 regulates its partnership with XPD

**Fig. 2.**
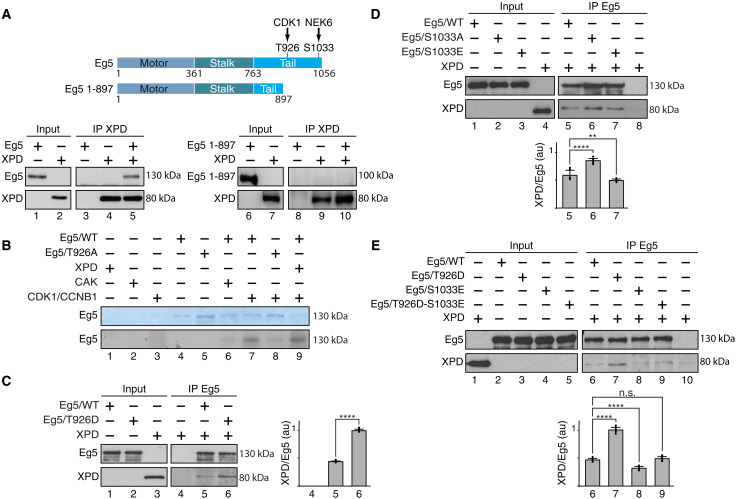
Phosphorylation modulates the partnership of Eg5 with XPD. (**A**) Schematic representation of the entire 1056–amino acid Eg5 protein (with the Motor, Stalk, and Tail domains) and the truncated form Eg5/1-897; the residues T926 and S1033 (which are phosphorylated by CDK1 and NEK6, respectively) are also indicated. Immunoprecipitated XPD (IP XPD) was incubated with either entire Eg5 or Eg5/1-897, and after washes, the coimmunoprecipitated proteins were resolved by SDS-PAGE and blotted with anti-XPD and anti-Eg5. (**B**) Purified Eg5/WT and Eg5/T926A were incubated (as indicated, +) with recombinant XPD, CAK (CDK7/cyclin H/MAT1), and CDK1/CCNB1 in the presence of [γ-^32^P]ATP (0.14 μM). Coomassie blue–stained gel (top) and the corresponding autoradiography (bottom) are shown. (**C** to **E**) When indicated (+), immunoprecipitated Eg5/WT, Eg5/T926D, Eg5/S1033A, Eg5/S1033E, or Eg5/T926D-S1033E was incubated with purified XPD/WT. After washes, the coimmunoprecipitated proteins were resolved by SDS-PAGE and blotted with anti-Eg5 and anti-XPD. The immunoprecipitated signals (IP) for XPD and Eg5 were quantified (*n* = 3, means ± SD), and the ratio XPD/Eg5 were plotted in arbitrary units (au). **P < 0.01 and ****P < 0.0001, Student’s *t* test; n.s., not significant.

To further understand how XPD and Eg5 interact, we generated a truncated form of Eg5 (1-897), in which its C-terminal part has been deleted. Immunoprecipitation assays showed that the Eg5 1-897 truncated form no longer interacted with recombinant XPD (Fig. 2A, lanes 6 to 10), suggesting that the C-terminal part of Eg5 (amino acids 898 to 1056) is essential for the XPD/Eg5 partnership. Knowing that the C-terminal part of Eg5 is subjected to phosphorylations (*15*), we wondered whether Eg5 might also be phosphorylated by CDK7 (as a subunit of the CAK module, which can be associated to XPD in the absence of core-TFIIH) (*4*–*6*). In vitro kinase assays showed that CAK was unable to phosphorylate Eg5/WT (Fig. 2B, lane 6). On the contrary, Eg5/WT was phosphorylated by the CDK1 [associated to cyclin B1 (CCNB1)] (lane 7), a posttranslational modification observed during mitosis (*19*, *20*). Accordingly, Eg5/T926A, in which the threonine residue T926 has been mutated into alanine, was not targeted by CDK1 (lane 8). We next observed that the presence of XPD did not modify the ability of CDK1 to phosphorylate Eg5 at T926 (compare lanes 7 and 9). We also investigated whether phosphorylation of Eg5 at T926 might affect its partnership with XPD. Notably, Eg5/T926D, in which the threonine-to-aspartate substitution mimics a constitutive phosphorylation (*21*), enhanced its binding with XPD relative to Eg5/WT (Fig. 2C, lanes 5 and 6).

Since Eg5 is also targeted by the kinase NEK6 at the serine residue S1033 (a phosphorylation required for proper mitotic spindle formation) (*22*), coimmunoprecipitation assays were performed with purified XPD and different recombinant Eg5 proteins. We first observed that the nonphosphorylatable form Eg5/S1033A had a moderately increased capacity to interact with XPD/WT relative to Eg5/WT and the phosphomimetic form Eg5/S1033E (Fig. 2D, compare lanes 5 to 7). The simultaneous presence of the phospho-mimetic S1033E and T926D mutations (resulting in Eg5/T926D-S1033E), circumvented the stimulatory effect of the T926D substitution (Fig. 2E, lanes 7 and 9), suggesting that the phosphorylation status of Eg5 regulates its partnership with XPD.

### Mutations found in patients with XP-D alter XPD/Eg5 partnership

**Fig. 3.**
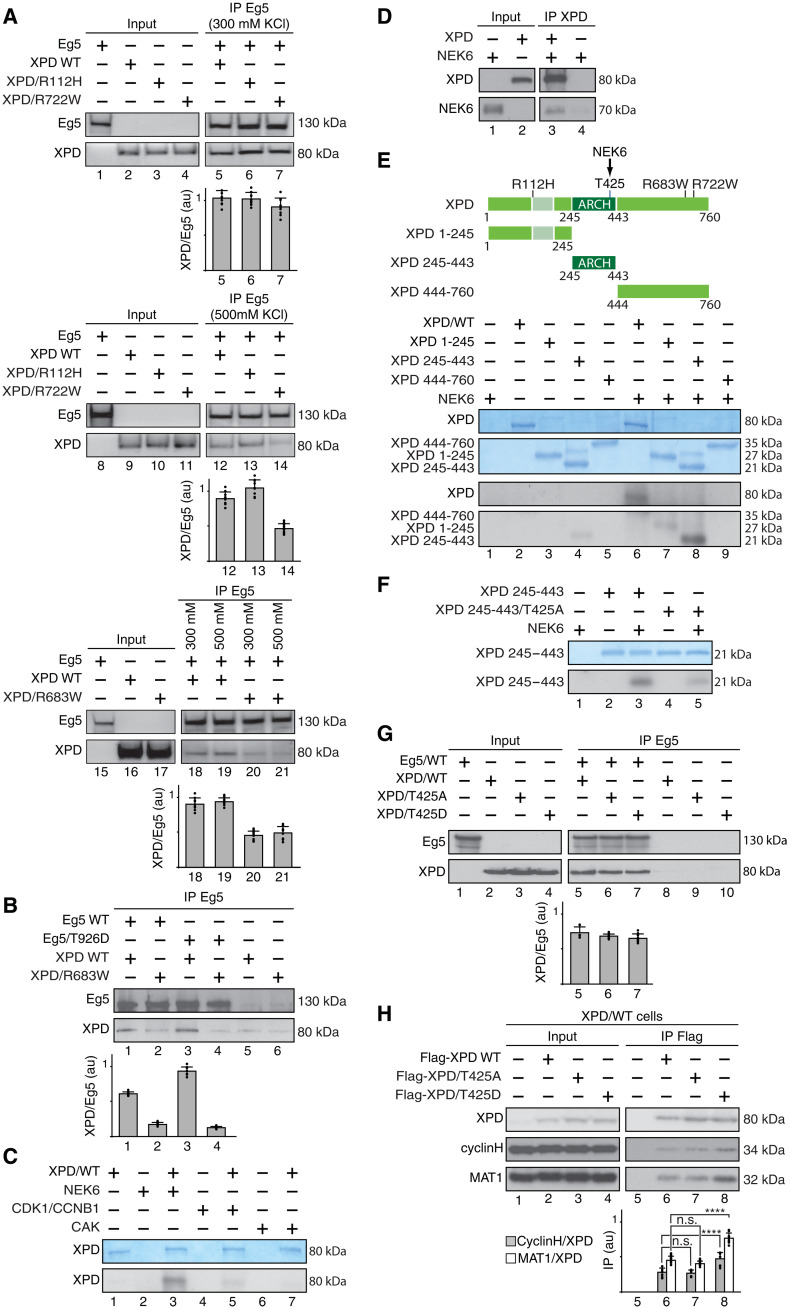
Phosphorylation of Eg5 regulates its partnership with XPD. (**A**) Coimmunoprecipitation at either 300 or 500 mM salt concentration of Eg5 (IP Eg5) with either XPD/WT, XPD/R112H, XPD/R722W, or XPD/R683W. The ratio XPD/Eg5 were plotted in au (*n* = 3, means ± SD). (**B**) Immunoprecipitated Eg5/WT and Eg5/T926D were incubated with either XPD/WT or XPD/R683W. The graph shows the ratio XPD/Eg5 (*n* = 3, means ± SD) in au. (**C**) Purified XPD was incubated with either recombinant NEK6, CDK1/CCNB1, or CAK (CDK7, cyclin H, and MAT1) in the presence of [γ-^32^P]ATP (0.14 μM). Coomassie blue–stained gel containing XPD (top) and the corresponding autoradiography (bottom) are shown. (**D**) Flag-XPD (IP XPD) was immunoprecipitated and incubated with tagged glutathione *S*-transferase (GST)–NEK6. As control, anti-Flag magnetic beads were incubated with GST-NEK6 alone. (**E**) Entire XPD and its truncated forms 1-245, 245-443, and 444-760 were incubated with NEK6 in the presence of [γ-^32^P]ATP (0.14 μM). Coomassie blue–stained gels and the corresponding autoradiographies are shown. (**F**) The ARCH domain (XPD 245-443) and its mutated form (XPD 245-443/T425A) were incubated with NEK6 in the presence of [γ-^32^P]ATP (0.14 μM). Coomassie blue–stained gel and the corresponding autoradiography are shown. (**G**) Coimmunoprecipitation of Eg5 (IP Eg5) with either XPD/WT, XPD/T425A, or XPD/T425D. The graph shows the ratio XPD/Eg5 (*n* = 3, means ± SD) in au. (**H**) Coimmunoprecipitation assays with whole-cell extracts isolated from XPD/WT cells overexpressing either Flag-XPD/WT, Flag-XPD/T425A, or Flag-XPD/T425D. The graph shows the ratio cyclin H/XPD (gray bars) and MAT1/XPD (open bars; *n* = 3, means ± SD) in au. ****P < 0.0001, Student’s *t* test.

We next wondered whether mutations found in patients with XP-D might disturb XPD/Eg5 partnership. The mutations XPD/R112H, XPD/R683W, and XPD/R722W were selected for their location in either the N-terminal (/R112H) or the C-terminal (/R683W and /R722W) part of XPD, their loss-of-function evolutionary effect (which is mixed for /R112H and /R722W and severe for /R683W) (*23*), and their association with either TTD (XPD/R112H and /R722W) or the cancer-prone disease XP (XPD/R683W). Purified mutant XPD proteins were first incubated with immunoprecipitated Eg5 (Fig. 3A). After washing at either 300 or 500 mM salt concentration, XPD/R722W (lanes 7 and 14) and XPD/R683W (lanes 20 to 21) interacted much less with Eg5 when compared to that obtained with XPD/WT (lanes 5, 12, 18, and 19). The partnership of Eg5 with XPD/R112H was similar to that observed with XPD/WT (compare lanes 5, 6, 12, and 13), suggesting that mutations located in the C-terminal part of XPD are detrimental for its interaction with Eg5. Since phosphorylation at T926 of Eg5 promoted its interaction with XPD/WT (Fig. 2C), we next investigated whether this phosphorylation modulated the ability of Eg5 to interact with XPD/R683W. However, contrary to Eg5/WT, Eg5/T926D did not modify its interaction with XPD/R683W (Fig. 3B, compare lanes 2 and 4).

Knowing that TFIIH subunits (especially CDK7 and XPB) can be posttranslationally modified (*24*, *25*), we examined whether any phosphorylation of XPD might modulate its interaction with Eg5. We primarily analyzed whether XPD might be phosphorylated by kinases targeting Eg5, namely, CDK1/CCNB1 and NEK6. In vitro kinase assays revealed that XPD was not phosphorylated by CDK1 (associated to CCNB1; Fig. 3C, lane 5) and CDK7 (within CAK, lane 7). However, NEK6 turned out to phosphorylate XPD (lane 3). Direct interaction was observed between recombinant XPD and NEK6 (Fig. 3D, lane 3), and confocal immunofluorescence microscopy analysis showed colocalization of XPD and NEK6 at the mitotic spindle and spindle poles in the early mitotic stages of prometaphase and metaphase (fig. S2A, images A.1 to A.4 and A.5 to A.8). Further experiments showed that contrary to the N- (XPD 1-245, lane 7) and the C-terminal (XPD 444-760, lane 9) part of XPD, the ARCH domain of XPD was notably phosphorylated by NEK6 (Fig. 3E, lane 8). Liquid chromatography coupled to tandem mass spectrometry (LC-MS/MS) next identified the threonine residue T425 as a potential target for NEK6. As a control, we observed a reduction for the phosphorylation by NEK6 of recombinant XPD 245-443 carrying the T425A mutation (Fig. 3F, lanes 3 and 5), although residual phosphorylation was still occurring because of the targeting of a residue not identified by LC-MS/MS or nonspecific phosphorylation following the substitution of the main phosphorylatable residue T425.

Since the phosphorylation status of Eg5 conditioned its partnership with XPD (Fig. 2, C to E), we then studied what might be the consequences of XPD phosphorylation on its interaction with Eg5. Coimmunoprecipitation assays showed that recombinant XPD/WT, XPD/T425A, and XPD/T425D interacted similarly with purified Eg5 (Fig. 3G), suggesting that the phosphorylation of XPD at T425 did not influence its interaction with Eg5. However, coimmunoprecipitation assays using whole-cell extracts isolated from HeLa (XPD/WT) cells overexpressing either Flag-tagged XPD/WT, XPD/T425A, or XPD/T425D revealed a stronger interaction of the CAK with XPD/T425D relative to that with XPD/WT and XPD/T425A (Fig. 3H, lanes 6 to 8). The interaction with other factors known to bind the ARCH domain (amino acids 245 to 443) of XPD, such as MMS19 (*26*) and the XPG NER endonuclease (*27*, *28*), was not modified by the XPD phosphorylation at T425 (fig. S2, B and C, respectively). Furthermore, the interaction of core-TFIIH with XPD/WT was not changed in the presence of either XPD/T425A or T425D (fig. S2D).

### Mitosis is perturbed in XP-D patient cells

**Fig. 4.**
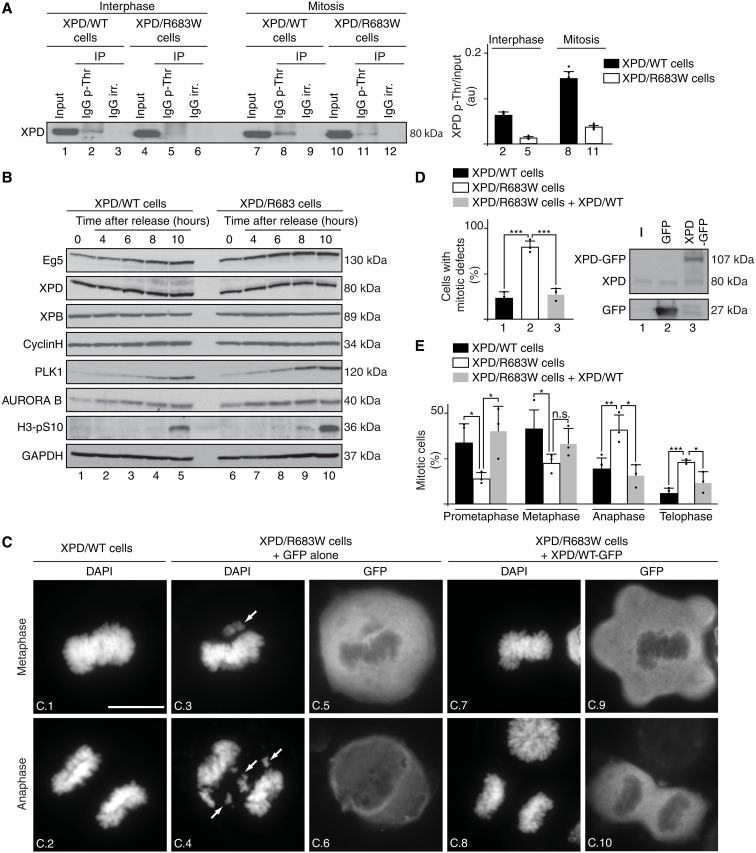
Mitosis is disrupted in XP-D patient cells. (**A**) Whole-cell extracts were isolated from XPD/WT and XPD/R683W cells in interphase and mitosis (upon nocodazole treatment and release for 90 min). Immunoprecipitations were performed with either irrelevant IgG (IgG irr.) or anti–phospho-threonine IgG (IgG p-Thr). The graph shows the ratio phosphorylated XPD (XPD p-Thr)/input in normal and XPD-mutated cells (*n* = 3, means ± SD) in au. (**B**) Western blot analysis (*n* = 3) of XPD/WT- and XPD/R683W-mutated cells synchronized with double thymidine block, released and collected at 0, 4, 6, 8, and 10 hours. glyceraldehyde-3-phosphate dehydrogenase (GAPDH) was used as loading control. (**C** and **D**) XPD/WT cells and XPD/R683W cells overexpressing the tag GFP alone or GFP-XPD/WT were analyzed in mitosis (upon nocodazole treatment and release for 90 min). The arrows point to chromosome segregation errors at metaphase and anaphase. Scale bar, 5 μm. (**D**) Percentage of mitotic cells displaying either a normal or a defective mitotic phenotype (at least 130 cells per experiment and per condition were counted). *n* = 3, means ± SD; ***P < 0.001, Student’s *t* test). Western Blots show overexpressed Tag GFP and XPD/WT-GFP. (**E**) XPD/WT cells (black bars) and XPD/R683W cells overexpressing either tag GFP alone (open bars) or GFP-XPD/WT (gray bars) were analyzed in mitosis (upon nocodazole treatment and release for 90 min). The data are shown as percent mitotic cells in each mitotic stage compared to the total cell number counted (*n* = 3, means ± SD; at least 130 cells per experiment and per condition were counted; *P < 0.05, **P < 0.01, and ***P < 0.001, Student’s *t* test).

We then analyzed whether XPD phosphorylation might be altered in XP-D patient cells. Antibodies against phospho-threonine were used to immunoprecipitate phosphorylated proteins from whole-cell extracts of XPD/WT (HeLa) and XPD/R683W (HD2) cells (*29*, *30*), at interphase and mitosis. Western blot analysis revealed a higher XPD phosphorylation rate in XPD/WT cells during mitosis than during interphase (Fig. 4A, compare lanes 2 and 8; see also quantification, right). Notably, the XPD phosphorylation was reduced both during interphase and mitosis in XP-D/R683W cells when compared to that observed in XPD/WT cells (compare lanes 2 and 5, as well as lanes 8 and 11). Furthermore, XPD phosphorylation weakly increased between interphase and mitosis in XPD/R683W cells (compare lane 5 to lane 11).

These observations prompted us to determine the fate of XPD and Eg5 along the cell cycle. Western blot analysis of cells synchronously progressing through mitosis revealed that the level of XPD (as well as of the XPB and cyclin H subunits of TFIIH) did not change along the mitotic time course and was nearly similar in XPD/WT and XPD/R683W cells (Fig. 4B, lanes 1 to 5 and 6 to 10, respectively). No differences were observed between XPD/R683W and XPD/WT cells for the accumulation of Eg5 or several established mitotic markers such as Polo-like kinase 1 (which triggers G_2_-M transition and establishes bipolar spindle) (*31*), AURORA B (which controls condensation of the chromosomes and their attachment to the mitotic spindle) (*32*), and H3-pS10 (the mitotic marker phospho–histone H3 serine-10). Immunofluorescence microscopy analysis of XPD/R683W cells (fig. S3A) revealed that the localization of mutated XPD during mitosis did not notably differ from what had been previously observed in XPD/WT cells (Fig. 1A), with a fraction of XPD that still colocalized with Eg5 at the mitotic spindle (fig. S3A, images A.10 to A.12, A.14 to A.16, and A.18 to A.20) and at the midbody (images A.22 to A.24).

Notably and contrary to XPD/WT cells, XPD/R683W cells displayed a large variety of severe mitotic defects, including misaligned chromosomes at metaphase and lagging chromosomes and chromatin bridges at anaphase (Fig. 4C, images C.3 and C.4). The total number of XPD/R683W cells with abnormal mitotic phenotype was significantly increased (79% versus 23%) relative to XPD/WT cells (Fig. 4D, open box and black box, respectively). Although protein overexpression can be harmful to the cells and have limiting effects (*33*), we observed that overexpression of tagged XPD/WT–green fluorescent protein [GFP; as verified by Western blots, Fig. 4D (right); by immunofluorescence, Fig. 4C (images C.9 and C.10)] rescued the chromosome segregation errors in XPD/R683W-mutated cells (images C.7 and C.8; Fig. 4D, gray box); note that the presence of the GFP tag did not interfere with the ability of XPD to interact with Eg5, as verified by coimmunoprecipitation assays (fig. S3B). Furthermore, we noticed that the distribution of mitotic stages (Fig. 4E) was markedly different in XPD/R683W cells (open boxes) when compared to XPD/WT cells (black boxes): reduction of the population of XPD/R683W cells in prometaphase (14% versus 34%) and metaphase (22% versus 41%) and increase in anaphase (41% versus 19%) and telophase (23% versus 6%). Overexpression of tagged XPD/WT-GFP restored the distribution of mitotic stages in XPD/R683W cells (gray boxes), highlighting the deleterious effects of the XPD/R683W mutation in mitosis.

### Phosphorylation of XPD is required for proper mitosis

**Fig. 5.**
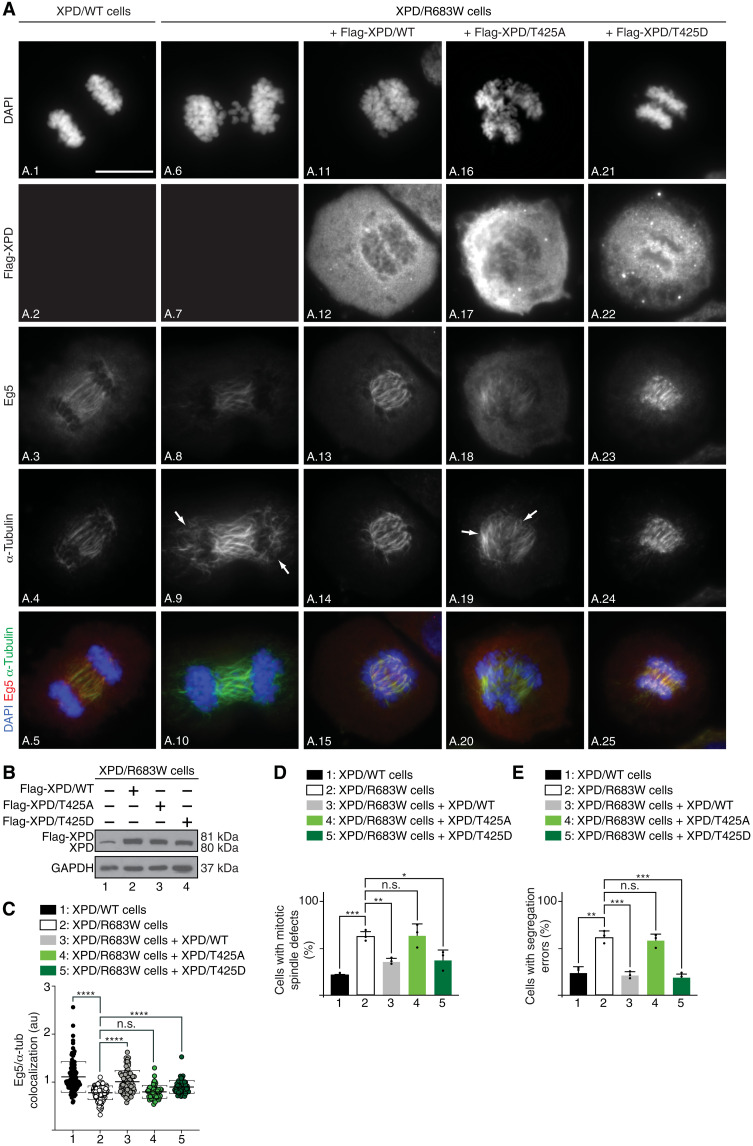
Phosphorylation of XPD is critical for mitosis. (**A**) Immunofluorescence of XPD/WT and XPD/R683W cells in anaphase overexpressing either Flag-XPD/WT, Flag-XPD/T425A, or Flag-XPD/T425D. Cells were synchronized in mitosis for 16 hours with nocodazole (100 ng/ml) and collected 90 min after nocodazole release. Immunofluorescence analyses were performed with antibodies targeting the Flag-Tag, Eg5, and α-tubulin. Chromosomes were stained with DAPI. The arrows point to mitotic spindle defects. Scale bar, 5 μm. (**B**) Overexpression of Flag-XPD/WT (lane 2), Flag-XPD/T425A (lane 3), and Flag-XPD/T425D (lane 4) in XPD/R683W cells were analyzed by Western blots. The level of endogenous XPD can be visualized in nontransfected XPD/R683W cells (lane 1). (**C**) Relative presence of Eg5 on the mitotic spindle [n = 3, means ± SD; two-tailed Student’s *t* test for sample 1 versus 2 and ordinary one-way analysis of variance (ANOVA) test for sample 2 versus 3, sample 2 versus 4, or sample 2 versus 5; ****P < 0.0001]. (**D** and **E**) Percentage of cells displaying mitotic spindle defects (D) and with segregation errors (E) (*n* = 3, means ± SD; at least 300 cells per experiment and per condition were counted; *P < 0.05, **P < 0.01, and ***P < 0.001, Student’s *t* test).

We next determined whether XPD phosphorylation regulates Eg5 localization and mitotic spindle assembly. Synchronized XPD/WT and XPD/R683W cells were subjected to immunofluorescence microscopy with antibodies targeting Eg5 and α-tubulin, which marks the formation and maintenance of the mitotic spindle as a readout for Eg5 activity (*15*). When compared to XPD/WT (Fig. 5A, images A.1 to A.5), the XPD/R683W cells (images A.6 to A.10) displayed various defective spindle phenotypes, such as bundled microtubules and unfocused spindle poles [image A.9; quantification, Fig. 5D (bar 2)], which were accompanied by a reduced Eg5 localization at the mitotic spindle (Fig. 5A, image A.8; Fig. 5C, plot 2). Defective Eg5 localization on microtubules and the associated mitotic spindle phenotypes were rescued upon XPD/WT overexpression [Fig. 5A (images A.11 to A.15); Fig. 5B (lane 2); Fig. 5, C and D (plot 3 and bar 3)]. Notably, Eg5 localization and consequent mitotic spindle defective phenotypes were restored in XPD/R683W cells upon overexpression of the phosphomimetic form XPD/T425D [Fig. 5A (images A.21 to A.25); Fig. 5B (lane 4); Fig. 5, C and D (plot 5 and bar 5)] but not of the nonphosphorylatable XPD/T425A [Fig. 5A (image A.16 to A.20); Fig. 5B (lane 3); Fig. 5, C and D (plot 4 and bar 4)]. Furthermore, while the XPD/R683W cells exhibited a higher rate of chromosome segregation errors than XPD/WT cells (Fig. 5E, bars 1 and 2), overexpression of either XPD/WT or XPD/T425D reduced these mitotic phenotypes (bars 3 and 5); cytokinesis failure detected in XPD/R683W cells was also reduced upon overexpression of either XPD/WT or XPD/T425D (fig. S4A, compare images A.6 to A.10 with images A.11 to A.15 and A.21 to A.25). No rescue was observed upon overexpression of XPD/T425A [Fig. 5E (bar 4) and fig. S4A (images A.16 to 20)]. Together, these results strongly suggest that the XPD phosphorylation drives the proper localization of Eg5, the correct assembly of the mitotic spindle, and, in extenso, the faithful chromosome segregation.

**Fig. 6.**
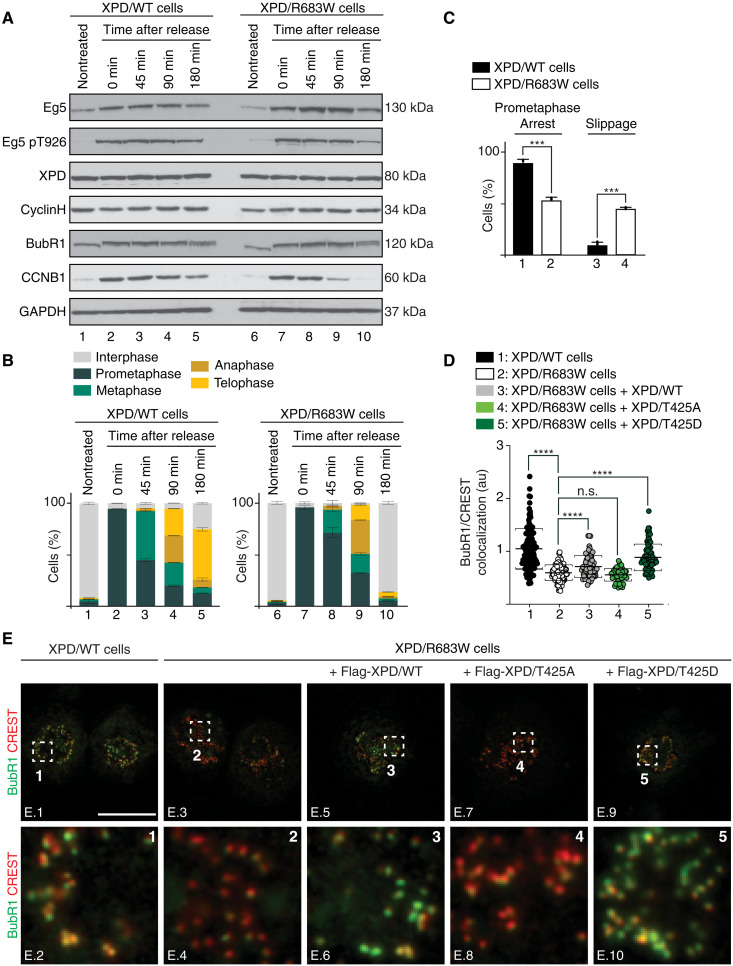
Mitotic slippage in XPD-mutated patient cells. (**A** and **B**) XPD/WT and XPD/R683W cells were synchronized with nocodazole, released, and harvested at indicated time points. (**A**) Whole-cell lysates were isolated and used for immunoblot analyses. GAPDH was used as loading control. (**B**) Fixed cells were mounted and stained with DAPI. The percentage of XPD/WT and XPD/R683W cells in different mitotic phases was quantified at the indicated time points after nocodazole release (*n* = 2, means ± SD; at least 200 to 250 cells were analyzed per condition and per experiment). (**C**) WT (black bars) and XPD/R683W (open bars) cells were treated with Taxol (16 hours, 1 μM). The percentage of cells arrested in prolonged prometaphase or exited mitosis was quantified (*n* = 3, means ± SD; at least 300 cells per experiment and per condition were counted; ***P < 0.001, Student’s *t* test). (**D** and **E**) WT and XPD/R683W cells overexpressing the Flag-Tag alone or fused to either XPD/WT, XPD/T425A, or XPD/T425D were synchronized in prometaphase with Taxol (16 hours, 1 μM). (**E**) Immunostaining of BubR1 and kinetochores (stained with CREST). Regions of interest are shown in the corresponding numbered panels. Scale bar, 5 μm. Unmerged images for Flag-XPD, BubR1, and CREST are provided in fig. S5B. (**D**) The relative intensity levels of BubR1 on individual kinetochores were quantified by using the Fiji software (*n* = 3, means ± SD; two-tailed Student’s *t* test for sample 1 versus 2 and ordinary one-way ANOVA test for sample 2 versus 3, sample 2 versus 4, or sample 2 versus 5; ****P < 0.0001).

To further dissect the mitotic role of XPD, XPD/WT and XPD/R683W cells were synchronized in prometaphase with nocodazole (16 hours, 100 ng/ml), washed out, and then collected at different time points. Western blot analysis revealed that the level of XPD and cyclin H did not change along the time course and was similar in XPD/WT and XPD/R683W cells (Fig. 6A). Eg5 accumulated at *t* = 0 in XPD/WT (Fig. 6A, lane 2) and XPD/R683W (lane 7) cells to then decrease at the end of the time course (lanes 3 to 5 and 8 to 10). The phosphorylation of Eg5 at T926 by CDK1 followed the premature degradation of CCNB1 in XPD/R683W cells (compare lanes 4 and 5 and 9 and 10). In sharp contrast to XPD/WT cells, the prometaphase-arrested XPD/R683W cells slightly progressed through mitosis 45 and 90 min after nocodazole release (Fig. 6B, compare bars 3 and 4 and 8 and 9), to finally reach interphase at 180 min, while a large majority of XPD/WT cells were still undergoing mitosis (compare bars 5 and 10), suggesting that XPD/R683W cells might exit mitosis prematurely.

We then checked whether the SAC, a process known to control the proper segregation of chromosomes during mitosis (*34*), might be defective in XPD/R683W cells. XPD/WT and XPD/R683W cells were treated with Taxol, which stabilizes microtubules and blocks metaphase to anaphase transition due to SAC activation. Such treatment induced prolonged mitotic arrest in XPD/WT cells (Fig. 6C, box 1), while in contrast, a significant number of XPD/R683W cells exited Taxol-induced mitotic arrest (box 4), displaying chromosome segregation errors (fig. S5A). The localization of the key SAC component BubR1 (budding uninhibited by benzimidazoles related 1) (*35*) to kinetochores [labeled with serum from patients with CREST (calcinosis, Raynaud phenomenon, esophageal dismotility, scierodactyly, telangiectasia) scleroderma] was significantly reduced in XPD/R683W cells (Fig. 6D, plot 2; Fig. 6E, images E.3 and E.4; fig. S5B, images B.9 to B.16). Notably, the total BubR1 protein levels remained unchanged between XPD/WT and XPD/R683W cells (Fig. 6A). The BubR1 localization to kinetochores was restored upon overexpression of either XPD/WT or XPD/T425D (Fig. 6D, plots 3 and 5; Fig. 6E, images E.6 and E.10; fig. S5B, images B.17 to B.24 and B.33 to B.40) but not of XPD/T425A (Fig. 6D, plot 4; Fig. 6E, image E.8; fig. S5B, images B.25 to B.32), suggesting that XPD phosphorylation is critical to maintain a functional mitotic checkpoint and to ensure correct chromosome segregation.

### Eg5/S1033A circumvents mitotic defects in XP-D cells

**Fig. 7.**
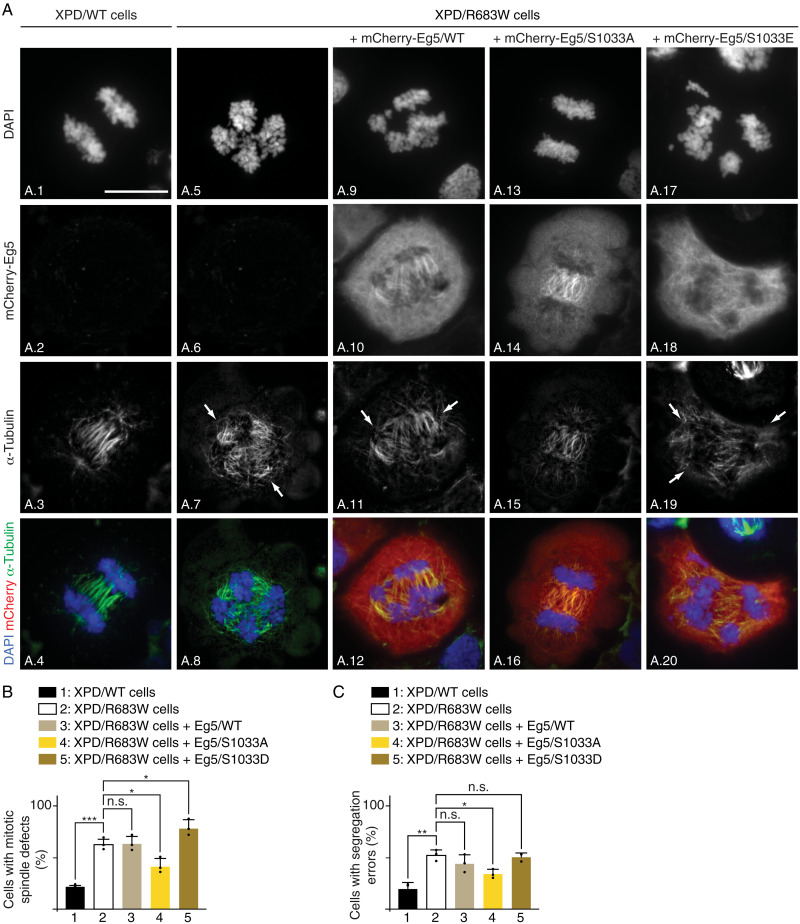
Eg5/S1033A restores mitotic defects in XPD-mutated patient cells. (**A**) Immunofluorescence of XPD/WT and XPD/R683W cells overexpressing either tagged mCherry-Eg5/WT, mCherry-Eg5/S1033A, or mCherry-Eg5/S1033E in anaphase. Cells were synchronized in mitosis for 16 hours with nocodazole (100 ng/ml) and collected 90 min after nocodazole release. Immunofluorescence analyses were performed with antibodies targeting the mCherry Tag and the mitotic spindle marker α-tubulin. Chromosomes were stained with DAPI. Arrows point to DNA bridges. Scale bar, 5 μm. (**B** and **C**) Percentage of cells displaying mitotic spindle defects (B) and segregation errors (C) (*n* = 3, means ± SD; at least 300 cells per experiment and per condition were counted; *P < 0.05, **P < 0.01, and ***P < 0.001, Student’s *t* test).

We also investigated whether the phosphorylation of Eg5 might, in turn, affect the mitotic phenotypes observed in XP-D cells. Unexpectedly, the overexpression of the nonphosphorylatable form Eg5/S1033A in XPD/R683W cells significantly rescued the mitotic spindle and chromosome segregation defects observed at anaphase [visualized by the α-tubulin marker and 4′,6-diamidino-2-phenylindole (DAPI) staining, respectively; Fig. 7A (compare image A.7 with A.15 and A.5 with A.13); quantification, Fig. 7, B and C (bars 2 and 4)] and at telophase (fig. S6A, images A.5 to A.8 and A.13 to A.16). On the contrary, overexpression of either Eg5/WT (which might therefore still be in vivo phosphorylated) or Eg5/S1033E did not modify the mitotic phenotypes resulting from XPD/R683W [Fig. 7A (images A.9 to A.12 and A.17 to A.20); Fig. 7, B and C (bars 3 and 5)]. Likewise, aberrant chromosome segregation and defective spindle formation observed at telophase were still present in XPD/R683W cells upon overexpression of either Eg5/WT or Eg5/S1033E (fig. S6A, images A.9 to A.12 and A.17 to A.20). Note that the tagged mCherry-Eg5/WT, /S1033A, and /S1033E proteins that were expressed at the same level, associated similarly to the endogenous Eg5 protein (fig. S6B). Furthermore, although having a lower capacity than XPD/WT to bind Eg5 (fig. S6C, compare bars 6 to 8 and 10 to 12), we observed that XPD/R683W exhibited a stronger interaction with Eg5/S1033A than with Eg5/S1033E (bars 11 and 12). Together, our data suggest that mitotic defects resulting from mutation of XPD can be restored by the nonphosphorylatable form S1033A of its partner Eg5.

### Phosphorylation of XPD is specifically required for its mitotic function

**Fig. 8.**
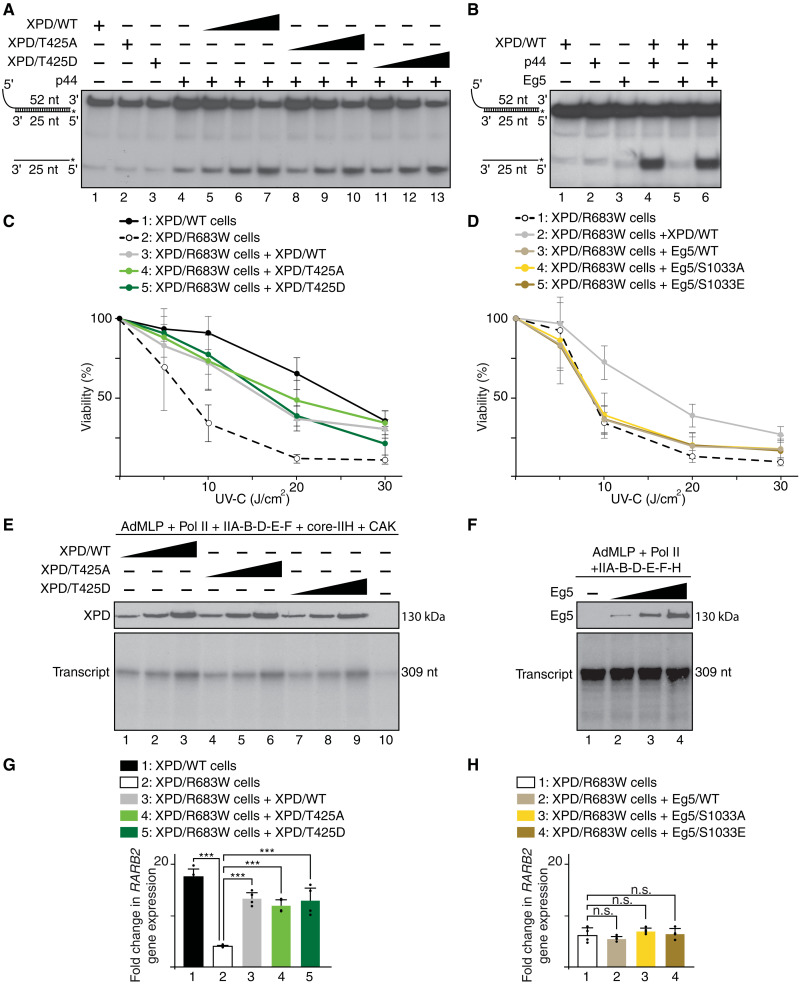
DNA repair and transcription do not require XPD phosphorylation. (**A** and **B**) Helicase assays in the presence (+) of p44 and Eg5 and increasing amounts of XPD/WT, XPD/T425A, and XPD/T425D. Single- and double-stranded DNA are indicated. (**C**) XPD/WT and XPD/R683W cells overexpressing XPD/WT, XPD/T425A, or XPD/T425D were treated with increasing doses of UV-C, and cell survival was determined 48 hours later. Data were normalized to unexposed cells (means ± SD of two experiments in triplicates). Significant statistical difference (Student’s *t* test): between XPD/WT and XPD/R683W cells at 5 J/cm^2^ (*P* < 0.001) and at 10, 20, and 30 J/cm^2^ (*P* < 0.0001); between XPD/R683W and XPD/R683W + XPD/WT or XPD/R683W + XPD/T425A cells at 10, 20, and 30 J/cm^2^ (*P* < 0.0001); and between XPD/R683W and XPD/R683W + XPD/T425D cells at 5 (*P* < 0.01), 10, and 20 J/cm^2^ (*P* < 0.0001) and at 30 J/cm^2^ (*P* < 0.001). (**D**) XPD/R683W cells overexpressing XPD/WT, Eg5/WT, Eg5/S1033A, or Eg5/S1033E were treated as indicated (C) (means ± SD of two experiments in triplicates). Significant statistical difference (Student’s *t* test) between XPD/R683W + XPD/WT cells and XPD/R683W, XPD/R683W + Eg5/WT, XPD/R683W + Eg5/S1033A, or XPD/R683W + Eg5/S1033E at 10, 20 (*P* < 0.0001), and 30 J/cm^2^ (*P* < 0.0001). (**E**) In vitro transcription assay with AdMLP template, RNAPII, TFIIA, TFIIB, TFIID(TBP), TFIIE, TFIIF core-IIH, CAK and increasing amounts of XPD/WT, XPD/T425A, or XPD/T425D. (**F**) Transcription assay with increasing amounts of Eg5. (**G**) *RAR*β*2* gene expression (normalized to the GAPDH RNA amount) after 8 hours of t-RA treatment in XPD/WT and XPD/R683W cells overexpressing XPD/WT, XPD/T425A, or XPD/T425D. The *RAR*β*2* mRNA expression is presented as *n*-fold induction relative to nontreated cells (means ± SD of two experiments in triplicates; ***P < 0.001, Student’s *t* test). (**H**) *RAR*β*2* gene expression in XPD/R683W cells overexpressing either Eg5/WT, Eg5/S1033A, or Eg5/S1033E (means ± SD of two experiments in triplicates).

We next were wondering whether the phosphorylation of XPD and Eg5, as well as their partnership, might affect the helicase activity of XPD, which is absolutely required during DNA repair (*2*). In the presence of the regulatory p44 subunit of TFIIH (which promotes the helicase activity of XPD) (*36*), XPD/T425A and XPD/T425D recombinant proteins exhibited the same helicase activity as XPD/WT [as observed in an in vitro assay by the displacement of the 5′ ^32^P-labeled 25–nucleotide (nt) single-stranded DNA annealed to the 52-nt single-strand DNA; Fig. 8A], suggesting that phosphorylation of XPD does not affect DNA repair activity. We also observed that addition of Eg5 modified neither the helicase activity of XPD (Fig. 8B) nor the removal of damaged oligonucleotides when added in an in vitro NER assay (containing cis-platinated DNA as a substrate, XPC, XPA, RPA, XPF/ERCC1, and XPG, as well as XPD, core-IIH, and CAK; fig. S7A). Knowing that patients with XPD mutations develop photosensitivity (*10*), we evaluated the cell viability upon UV irradiation. When compared to XPD/WT cells, XPD/R683W-mutated cells exhibited a lower survival rate, which was circumvented upon overexpression of either XPD/T425A, XPD/T425D, or XPD/WT (Fig. 8C). However, no restoration was detected upon overexpression of either XPD/R683W, XPD/R683W-T425A, or XPD/R683W-T425D (fig. S7B) and of Eg5/WT, Eg5/S1033A, and Eg5/S1033E (Fig. 8D). Together, the above data suggest that neither the phosphorylation of XPD nor that of Eg5 can rescue the deleterious effect of the XPD/R683W mutation in DNA repair.

We also analyzed whether the phosphorylation of XPD and its association with Eg5 might affect RNA synthesis. In vitro transcription assay [containing the adenovirus major late promoter (AdMLP) template, RNAPII, TFIIA, -IIB, -D(TBP), -E, -F, core-IIH, and CAK] showed that addition of either XPD/T425A or XPD/T425D promoted RNA synthesis with the same efficiency as XPD/WT (Fig. 8E). Furthermore, in vitro transcription was not altered by the addition of Eg5 (Fig. 8F). We also investigated the impact of XPD and Eg5 phosphorylation on the transactivation process by using the all-trans-retinoic acid (t-RA)–inducible *RAR*β*2* gene as a model (*37*). Overexpression of XPD/T425A and XPD/T425D restored similarly to XPD/WT the *RAR*β*2* gene expression in XPD/R683W cells (Fig. 8G), restoration that did not occur upon overexpression of XPD/R683W, XPD/R683W-T425A, and XPD/R683W-T425D (fig. S7C). The overexpression of either Eg5/S1033A, Eg5/S1033E, or Eg5/WT was unable to rescue the *RAR*β*2* expression deficiency observed in XPD/R683W cells (Fig. 8H), which was in accordance with the fact that Eg5 neither targeted nor influenced the formation of the transcription preinitiation complex (PIC; fig. S7D). Together, our results demonstrate that DNA repair and transcription are not dependent on the phosphorylation of both XPD and Eg5, which seems to be specifically required for mitosis.

## DISCUSSION

The present study aims to dissect the role of XPD in mitosis, beside its key functions in transcription and DNA repair as part of TFIIH. By establishing a phospho-dependent partnership with the motor kinesin protein Eg5, XPD critically regulates mitotic progression in a TFIIH-independent manner. XPD mutations lead to chromosome segregation errors, which might contribute to the development of clinical features observed in XP-D patients.

In human cells, the protein XPD can be found either associated to the CAK module, within TFIIH, or as a part of MMXD (*7*, *9*). XPD, independently of TFIIH and MMXD subunits (fig. S1, A and B), interacts and colocalizes with the motor kinesin Eg5, especially to mitotic spindle and midbodies (Fig. 1, A and B) (*16*). These observations raise questions on a potential mechanism regulating the ability of XPD to be either internalized within complex such as TFIIH or bound to other factors including Eg5. This switch mechanism might imply coordinated and sequential posttranslational modifications.

In this regard, we found that the mitotic kinase NEK6 (*38*) localizes with XPD in early phases of mitosis (fig. S2A) and phosphorylates the ARCH domain of XPD, at position T425 (Fig. 3E). This phosphorylation affects the ability of XPD to interact with the CAK module of TFIIH (Fig. 3H) but not with either Eg5 (Fig. 3G) or other factors such as MMS19, XPG, and the core-TFIIH (fig. S2, B to D) (*26*–*28*). Contrary to NEK6, neither CDK8 (a subunit of the transcription complex Mediator) (*24*, *39*), CDK7 (the kinase of the CAK module targeting transcription factors and mitotic CDKs) (*2*), nor CDK1 (a key mitotic kinase targeted by CDK7) (*40*) is able to phosphorylate XPD (Fig. 3C). Notably, we demonstrate that the phosphorylation of XPD at position T425 is critical for its mitotic functions (Figs. 5, A to E, and 6, D and E) without affecting its role in both DNA repair (Fig. 8, A and C) and transcription (Fig. 8, E and G). Besides the phosphorylation of XPD and given the obvious implication of XPD and Eg5 in mitosis, we subsequently observed that the phosphorylation of Eg5 is critical by modulating the Eg5/XPD partnership. While the Eg5/XPD interaction is promoted by the phosphorylation of Eg5 at T926 (Fig. 2C), it decreases when Eg5 is simultaneously phosphorylated at S1033 (Fig. 2E).

A better understanding of the phospho-dependent XPD/Eg5 partnership also came from studies of XPD-mutated forms. XPD mutations found in most patients with XP-D alter the ability of XPD to interact with Eg5 (Fig. 3A), which consequently disrupt the localization of Eg5 on microtubules, as observed in XPD/R683W-deficient cells (Fig. 5, A and C). This Eg5 mislocalization can be rescued by the expression of XPD/WT only if it is phosphorylatable at position T425 (Fig. 5). In addition, the overexpression of the nonphosphorylatable form Eg5/S1033A (which prevents XPD/Eg5 dissociation; fig. S6C) can circumvent mitotic failures (Fig. 7, A to C) but not DNA repair deficiencies (Fig. 8D) observed in XPD/R683W-mutated cells. These results suggest that the mitotic defects observed in XPD-mutated cells would not be related to defective DNA repair. Moreover, it even seems that functional mitosis might not require the DNA-dependent helicase activity of XPD (which is strongly reduced by the XPD/R683W mutation) (*41*). Although the mitotic function of XPD may not require its DNA-dependent helicase activity and its ability to bind DNA (illustrated by its chromosome exclusion in mitosis; Fig. 1A), it would be of interest to determine whether mitosis might be affected in cells bearing mutations (such as XPD/G47R) located within the ATP-binding site of XPD. Note that none of the transcription/DNA repair functions of XPD as a component of TFIIH are affected by Eg5 and this, whatever its phosphorylated status (Fig. 8, B, D, F, and H, and fig. S7, A and D). By highlighting the central role played by NEK6, our results suggest that phosphorylation process conditions the TFIIH-independent role of XPD in mitosis by acting as a switch mechanism to fine-tune its partnerships. Note that XPD is not the only component of TFIIH having mitotic functions. In particular, the CAK subcomplex (via CDK7) is known to phosphorylate and activate CDK1, which promotes entry into mitosis (*42*). Furthermore, TFIIH subunits (in particular, p52 and XPB) might be implicated in condensing and maintaining chromosome structure during mitosis in vertebrates (*43*) and *Drosophila* (*44*, *45*).

In addition, to underline the phospho-dependent mitotic function of XPD apart from its DNA repair/transcription functions, the present study provides insights into the understanding of the clinical features observed in patients with XP-D. In particular, by affecting the Eg5 localization and the microtubule organization (Fig. 5, A to E), the XPD/R683W mutation has deleterious consequences for mitosis, resulting in misaligned chromosomes, lagging chromosomes, and chromatin bridges (Fig. 4, C and D), which likely lead to cytokinesis defects (fig. S4A) (*46*). The chromosome segregation errors arise, in part, from defective SAC (or mitotic checkpoint) since the XPD-mutated cells bypass mitotic arrest by the microtubule poison Taxol (Fig. 6C) and prematurely exit mitosis (Fig. 6B), a process termed mitotic slippage (*47*) that is clearly illustrated by the premature degradation of CCNB1 (Fig. 6A). In addition to the genomic instabilities resulting from NER defect, the mitotic slippage evidenced here might contribute to the development of clinical features, especially the high risk of cancers commonly observed in patients with XP-D (*10*, *48*). In addition and knowing that processes other than mitosis require Eg5, such as growth and morphology of postmitotic neurons (*49*–*51*), defective action of Eg5 might contribute to neurological abnormalities of patients with XP-D. Since the clinical heterogeneity observed between patients with XP-D (*52*) might result from the combinatory effects of XPD-mutated forms (*53*), the effect of different mutated *XPD* on DNA repair, transcription, and mitosis process should be examined in suitable cellular models. Phenotypic recovery by Eg5 might be helpful to compensate the deleterious mitotic effects resulting from XPD mutations.

## MATERIALS AND METHODS

### Cell counting

Cells (at least 100 per condition and per experiment) were analyzed in a blinded manner for their chromosomal and mitotic spindle phenotypes. Normal and defective (chromosome misalignments, lagging chromosomes, anaphase/telophase bridges, and polylobed/abnormal nuclei) chromosomal phenotypes were assessed by DAPI staining. Normal and defective bipolar mitotic spindle formations were assessed by α-tubulin staining.

### Cell synchronization

Cells were synchronized in different stages of cell cycle by double thymidine block and release protocol. Briefly, 2 mM thymidine was added twice for 16 hours with an 8-hour interval in fresh medium between the thymidine treatments. After the second thymidine block was complete, cells were released in thymidine-free media and collected at the indicated time points. In addition, cells were synchronized in prometaphase using nocodazole (16 hours, 100 ng/ml) or Taxol (16 hours, 1 μM).

### Cell viability assays

Cells (50,000 cells per well in six-well petri dishes) were exposed to various doses of UV-C (predominantly 254 nm; Philips TUV lamp). After 48 hours, the cells were stained with 0.2% Crystal Violet (Sigma-Aldrich). After washing and drying, the stain was solubilized with 1% SDS and the optical density (595 nm) was measured.

### Helicase assays

The helicase assay probe (*27*) was incubated as indicated (+) with XPD (/WT, /T425A, or /T425D), p44, and Eg5, in the presence of ATP. Single- and double-stranded DNA were separated by electrophoresis and analyzed by autoradiography.

### Immunofluorescence microscopy

Once collected, the cells were centrifuged (on Thermo Scientific Shandon Cytospin 4 Cytocentrifuge, 5 min at 1000 rpm) and fixed [4% paraformaldehyde (PFA), 10 min]. After permeabilization (0.5% NP-40 for 5 min), the cells were washed [phosphate-buffered saline (PBS)–0.01% Triton], blocked (3% bovine serum albumin, 1 hour), and subsequently incubated with primary antibodies (see Key Resources Table) in blocking buffer. After washing (PBS–0.01% Triton), the corresponding secondary antibodies were added. Glass coverslips were then added on cells already mounted with either Mowiol (Calbiochem) or ProLong Gold Antifade agent (Invitrogen) containing DAPI. Images were taken with a ×63 objective using Zeiss epifluorescence microscope and/or confocal microscopy (Nikon spinning disk). Image analysis was performed using ImageJ software.

### Immunoprecipitation assays

Depending on the coimmunoprecipitation assays, WT and mutated forms of Eg5 and XPD were coincubated together with specific antibodies (as indicated) bound to protein G magnetic beads (Dynabeads, Invitrogen). After extensive washings, Western blots were performed using antibodies raised against the proteins of interest.

### Kinase assays

Highly purified proteins were incubated 30 min at 30°C with either recombinant CAK, CDK1/CCNB1, or NEK6 in the presence of [γ-^32^P]ATP (0.14 μM).

### LC-MS/MS analysis

Purified XPD-ARCH domain has been subjected to double digestion with trypsin and chymotrypsin. Samples were analyzed using an Ultimate 3000 nano-RSLC (Thermo Scientific) coupled in line with an LTQ-Orbitrap Elite mass spectrometer via a nanoelectrospray ionization source (Thermo Scientific). Peptides were identified by database searching using SequestHT (Thermo Fisher Scientific) with Proteome Discoverer 2.4 software (PD2.4, Thermo Fisher Scientific) on *Homo sapiens* database (Swiss-Prot; reviewed and released 6 April 2020; 20,286 entries). Precursor and fragment mass tolerances were set at 7 parts per million and 0.02 Da, respectively, and up to two missed cleavages were allowed. Oxidation (M) and phosphorylation (S/T/Y, +79.966) were set as variable modification.

### NER assays

Dual-incision assays were carried out in the presence of ATP (2 mM), plasmid (Pt-GTG) with single cisplatin adduct, purified XPC/hHR23B, core-TFIIH, CAK, XPD, XPA, RPA, XPG, XPF/ERCC1, and Eg5 (when indicated).

### PIC formation assays

Biotinylated AdMLP bound to streptavidin magnetic beads was incubated with RNAPII, TFIIA, TBP, TFIIB, TFIIF, TFIIE, core-IIH, CAK, XPD, and Eg5. After several washing, the bound fractions were resolved by SDS–polyacrylamide gel electrophoresis followed by immunoblotting (*54*).

### Plasmid transfections

Transient transfections of cDNAs encoding either mCherry alone, mCherry-Eg5/WT, mCherry-Eg5/S1033A, mCherry-Eg5/S1033E, GFP alone, XPD/WT-GFP, Flag-XPD/WT, Flag-XPD/T425A, or Flag-XPD/T425D were performed using X-tremeGENE9 (Roche) according to the manufacturer’s instructions.

### Purifications of recombinant proteins

The different forms of Flag-Eg5 (/WT, /1-897, /T926A, /T926D, /S1033A, /S1033E, and /T926D-S1033E) were produced in *Escherichia coli*. To purify Flag-MMS19, XPG, core-TFIIH (containing XPB, p62, p52, p44, p34, and p8), Flag-CAK (Flag-CDK7, cyclin H, and MAT1), and the different forms of Flag-XPD (/WT, /T425A, /T425D, /R112H, /R683W, /R722W, /1-245, /245-443, /245-443, 245-443/T425A, and /444-760), Sf21 insect cells were infected with the corresponding baculoviruses. The whole-cell extracts were then incubated with agarose beads bound to either anti-XPG (to immunoprecipitate XPG), anti-p44 (to immunoprecipitate core-TFIIH), or anti–M2-Flag (to immunoprecipitate Eg5, MMS19, CAK, and XPD) antibody. The recombinant proteins were then eluted with the corresponding epitope peptide.

### Reagents and resources

The reagents and resources (antibodies, chemical, cell lines, oligonucleotides, recombinant DNA, primers, software, and materials) used to accomplish this work are available in Key Resources Table (Supplementary Materials).

### Retrotranscription and real-time quantitative polymerase chain reaction

Total RNAs (2 μg) were reverse-transcribed by using Moloney murine leukemia virus RT and random hexanucleotides. FastStart DNA Master SYBR Green kit and the LightCycler apparatus (Roche Diagnostics) have been used to accomplish the real-time quantitative polymerase chain reaction. Primers are available in Key Resources Table (Supplementary Materials).

### Strength of the SAC response

Cells were treated with Taxol (16 hours, 1 μM) to induce prometaphase arrest. Cells were next collected by cytospin, fixed (4% PFA, 10 min), and washed three times with PBS. Fixed cells were mounted and stained with Mowiol containing DAPI (see Key Resource Table). The strength of the SAC response was monitored by counting the number of cells arrested in prometaphase versus the number of cells that displayed anaphase-like phenotypes based on their DNA morphology.

### Transcription assays

After preincubation of the AdMLP template with RNAPII, TFIIA, TBP, TFIIB, TFIIF, TFIIE, TFIIH, and Eg5, RNA synthesis was initiated by the addition of nucleoside triphosphate (200 μM), including radiolabeled cytidine triphosphate (0.15 μM) (*55*). The transcription activity was assessed after 20 min of incubation.

## Supplementary Materials

### This PDF file includes:

Figs. S1 to S7 Key Resources Table

## Notes

View/request a protocol for this paper from *Bio-protocol*.
