# Supplementary material for: Phosphorylation of XPD drives its mitotic role independently of its DNA repair and transcription functions
Source: Sci Adv. 2022 Aug 17;8(33):eabp9457. doi: 10.1126/sciadv.abp9457 (PMC9385140; doi:10.1126/sciadv.abp9457)
Supplement: Supplementary file 1 — Figs. S1 to S7 Key Resources Table [file sciadv.abp9457_sm.pdf]

Science Advances
AAAS

Supplementary Materials for

Phosphorylation of XPD drives its mitotic role independently of its DNA repair and transcription functions

Emmanuel Compe *et al.*

Corresponding author: Emmanuel Compe, compe@igbmc.fr

Sci. Adv. **8**, eabp9457 (2022)
DOI: 10.1126/sciadv.abp9457

The PDF file includes:

Figs. S1 to S7
Key Resources Table
Legend of source data file

Other Supplementary Material for this manuscript includes the following:

Source data file

Figure S1

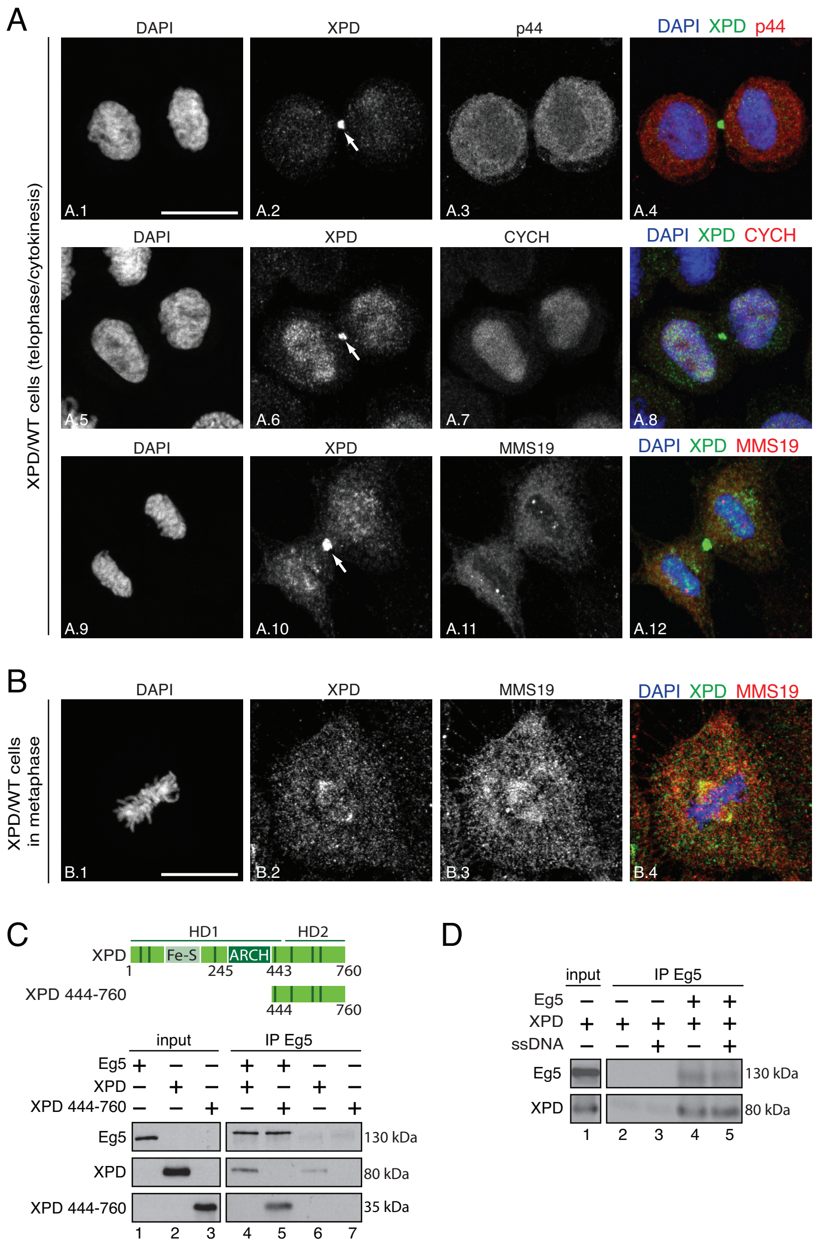

A
DAPI
XPD
p44
DAPI XPD p44
A.1
A.2
A.3
A.4
DAPI
XPD
CYCH
DAPI XPD CYCH
A.5
A.6
A.7
A.8
DAPI
XPD
MMS19
DAPI XPD MMS19
A.9
A.10
A.11
A.12
XPD/WT cells (telophase/cytokinesis)
B
DAPI
XPD
MMS19
DAPI XPD MMS19
B.1
B.2
B.3
B.4
XPD/WT cells in metaphase
C
HD1
HD2
XPD
Fe-S
ARCH
1
245
443
760
XPD 444-760
444
760
input
IP Eg5
Eg5 + – – + + – –
XPD – + – + – + –
XPD 444-760 – – + – + – +
Eg5
XPD
XPD 444-760
130 kDa
80 kDa
35 kDa
1 2 3 4 5 6 7
D
input
IP Eg5
Eg5 – – – + +
XPD + + + + +
ssDNA – – + – +
Eg5
XPD
130 kDa
80 kDa
1 2 3 4 5

Fig. S1. Immunolocalization of XPD and partners in XPD/WT cells.

(**A**) Localization of XPD, p44, Cyclin H and MMS19 during cytokinesis. XPD/WT cells were synchronized by double thymidine block, released in mitosis and analyzed by confocal immunofluorescence microscopy. Cells in telophase were identified according to their DAPI staining. The arrows point to the localization of XPD at the midbody Scale bar is 5μm.

(**B**) Localization of XPD and MMS19 during metaphase. XPD/WT cells were synchronized by double thymidine block, released in mitosis and analyzed by confocal immunofluorescence microscopy. Cells in metaphase were identified according to their DAPI staining. Scale bar is 5μm.

(**C**) Schematic representation of entire 760-aa XPD protein and the truncated form corresponding to the C-terminal part of XPD (444-760). Immunoprecipitated Eg5 (IP Eg5) was incubated with either entire Flag-XPD or Flag-XPD (444-760) fragment (as indicated, +). After washes, the coimmunoprecipitated proteins were resolved by SDS-PAGE and blotted with anti-Flag and Eg5. The results are representative of two independent experiments.

(**D**) Recombinant Eg5 and XPD were incubated in the presence of single-strand DNA (ssDNA, 7.5nM) and immunoprecipitated with anti-Eg5 (IP Eg5). After washes, coimmunoprecipitated proteins were resolved by SDS-PAGE and blotted with anti-Eg5 and -XPD. The results are representative of two independent experiments.

Figure S2

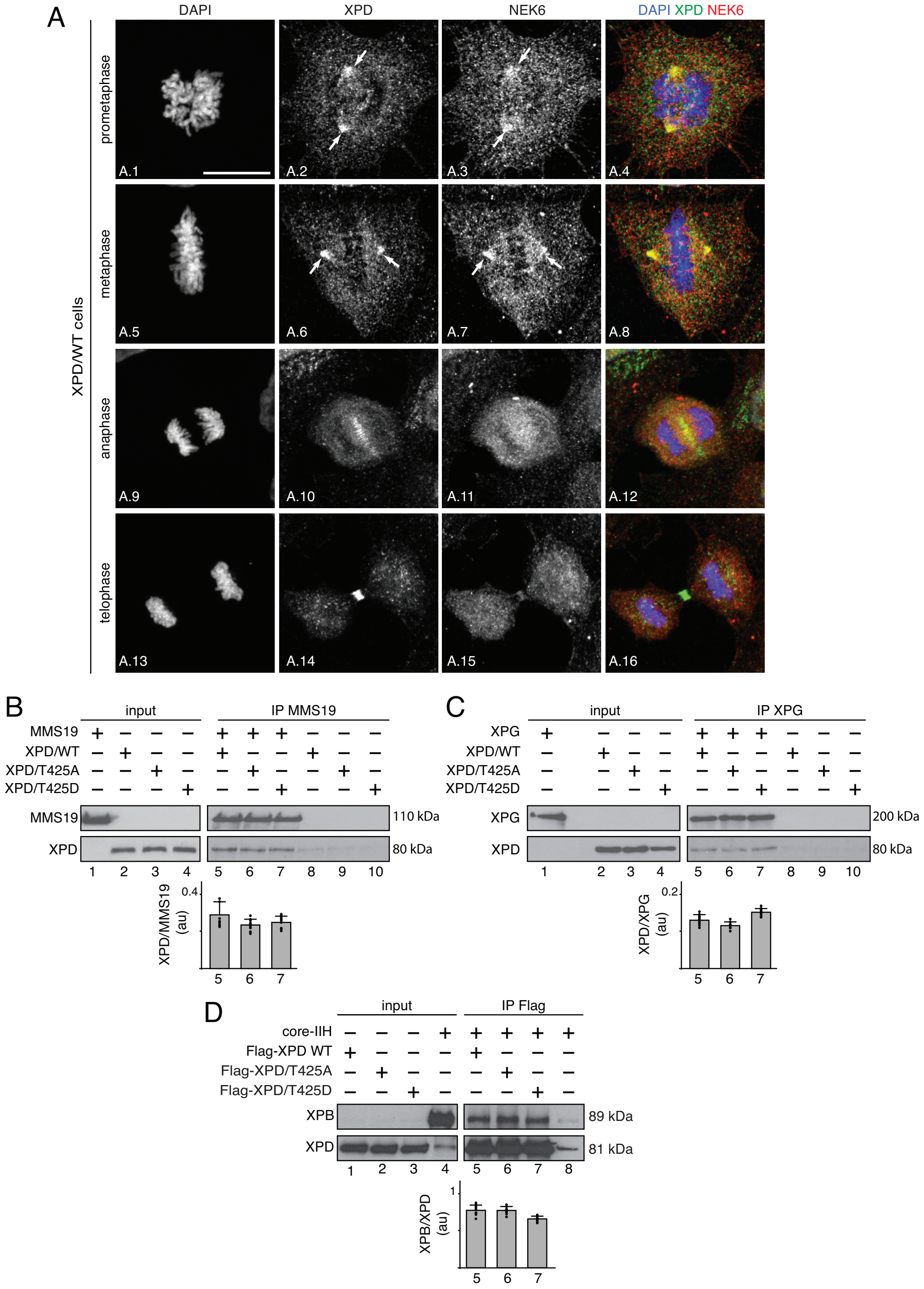
A
DAPI
XPD
NEK6
DAPI XPD NEK6
XPD/WT cells
prometaphase
metaphase
anaphase
telophase
A.1
A.2
A.3
A.4
A.5
A.6
A.7
A.8
A.9
A.10
A.11
A.12
A.13
A.14
A.15
A.16
B
input
IP MMS19
MMS19
XPD/WT
XPD/T425A
XPD/T425D
110 kDa
80 kDa
XPD/MMS19 (au)
0.4
C
input
IP XPG
XPG
XPD/WT
XPD/T425A
XPD/T425D
200 kDa
80 kDa
XPD/XPG (au)
0.2
D
input
IP Flag
core-IIH
Flag-XPD WT
Flag-XPD/T425A
Flag-XPD/T425D
XPB
XPD
89 kDa
81 kDa
XPB/XPD (au)
1

Fig. S2. Interaction of MMS19, XPG and core-TFIIH with XPD, depending on the XPD/T425 phosphorylation state.

(A) Immunolocalization of XPD and NEK6 during different mitotic phases. Human XPD/WT cells were synchronized by double thymidine block, released in mitosis and analyzed by confocal microscopy at prometaphase, metaphase, anaphase and telophase. The arrows point to the co-localization of XPD and NERK6 at the mitotic spindle and spindle poles in prometaphase and metaphase. Scale bar is 5µm.

(**B**-**C**) Immunoprecipitated MMS19 (IP MMS19, panel **B**) or XPG (IP XPG, panel **C**) was incubated with either XPD/WT, /T425A or /T425D. After washes, the co-immunoprecipitated proteins were resolved by SDS-PAGE and blotted with anti-XPD and either anti-MMS19 or XPG. Graph shows the ratio XPD/MMS19 (**B**) or XPD/XPG (**C**) (n=3, means±s.d.) in arbitrary units (au).

(**D**) Recombinant core-TFIIH (containing the p8, p34, p44, p52, p62, XPB) was incubated with either immunoprecipitated Flag-XPD/WT, /T425A or /T425D. After washes, the co-immunoprecipitated proteins were resolved by SDS-PAGE and blotted with anti-XPB and XPD. Graph shows the ratio XPD/XPB (n=3, means±s.d.) in arbitrary units (au).

Figure S3

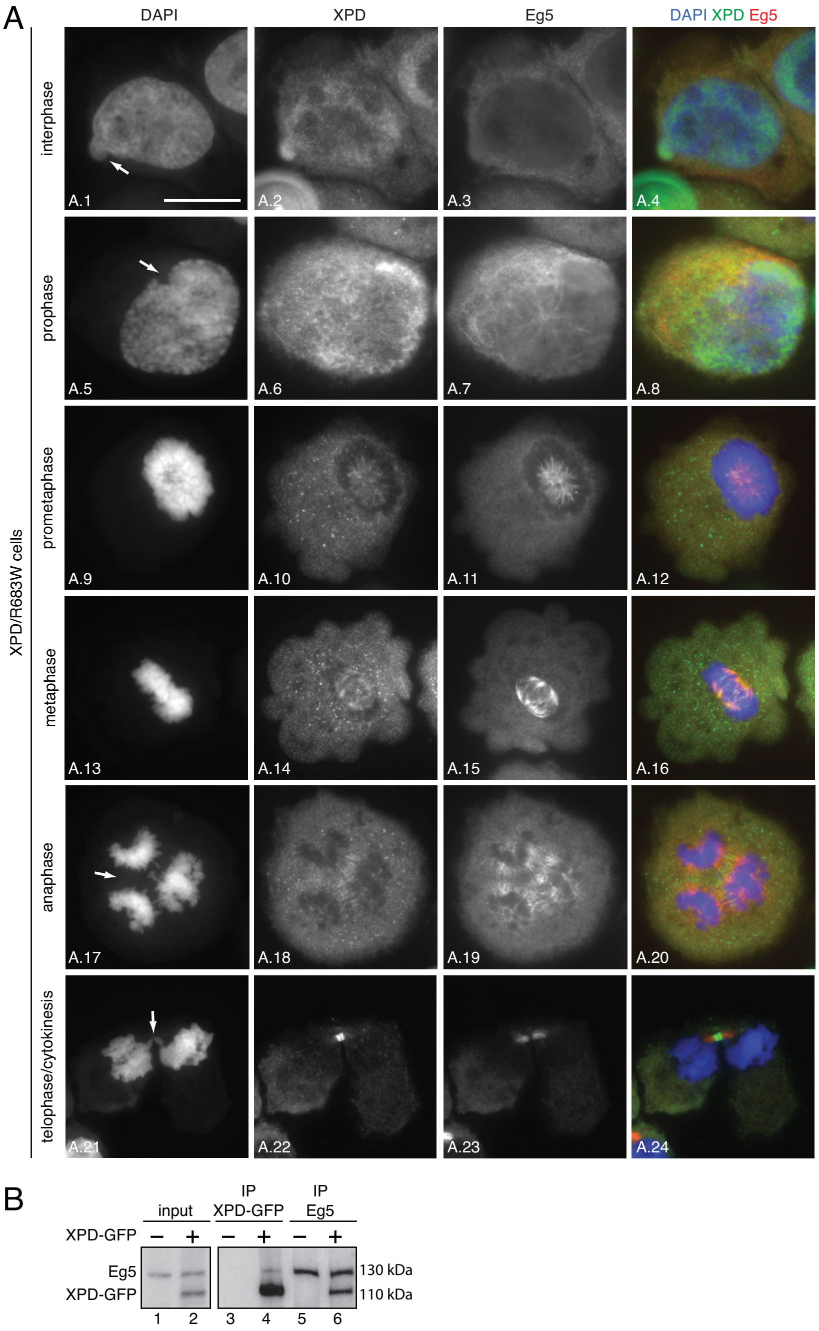
A
DAPI
XPD
Eg5
DAPI XPD Eg5
XPD/R683W cells
interphase
prophase
prometaphase
metaphase
anaphase
telophase/cytokinesis
A.1
A.2
A.3
A.4
A.5
A.6
A.7
A.8
A.9
A.10
A.11
A.12
A.13
A.14
A.15
A.16
A.17
A.18
A.19
A.20
A.21
A.22
A.23
A.24
B
input
IP XPD-GFP
IP Eg5
XPD-GFP
−
+
−
+
−
+
Eg5
XPD-GFP
130 kDa
110 kDa
1
2
3
4
5
6

Fig. S3. Immunolocalization of XPD and Eg5 in XPD/R683W cells.

(**A**) Immunofluorescence of XPD/R683W cells synchronized with double thymidine block and collected 9h after release. XPD and Eg5 localization was monitored through all the cell cycle stages (cells from each mitotic phase were identified according to their DAPI staining). The arrows point to irregular nuclear shapes and chromosome segregation errors. Scale bar is 5μm.

(**B**) Immunoprecipitation of either XPD (IP XPD-GFP) or Eg5 (IP Eg5) from cells overexpressing (when indicated, +) C-terminally GFP-tagged-XPD. After washes, the co-immunoprecipitated proteins were resolved by SDS-PAGE and blotted with antibodies targeting XPD and Eg5.

Figure S4

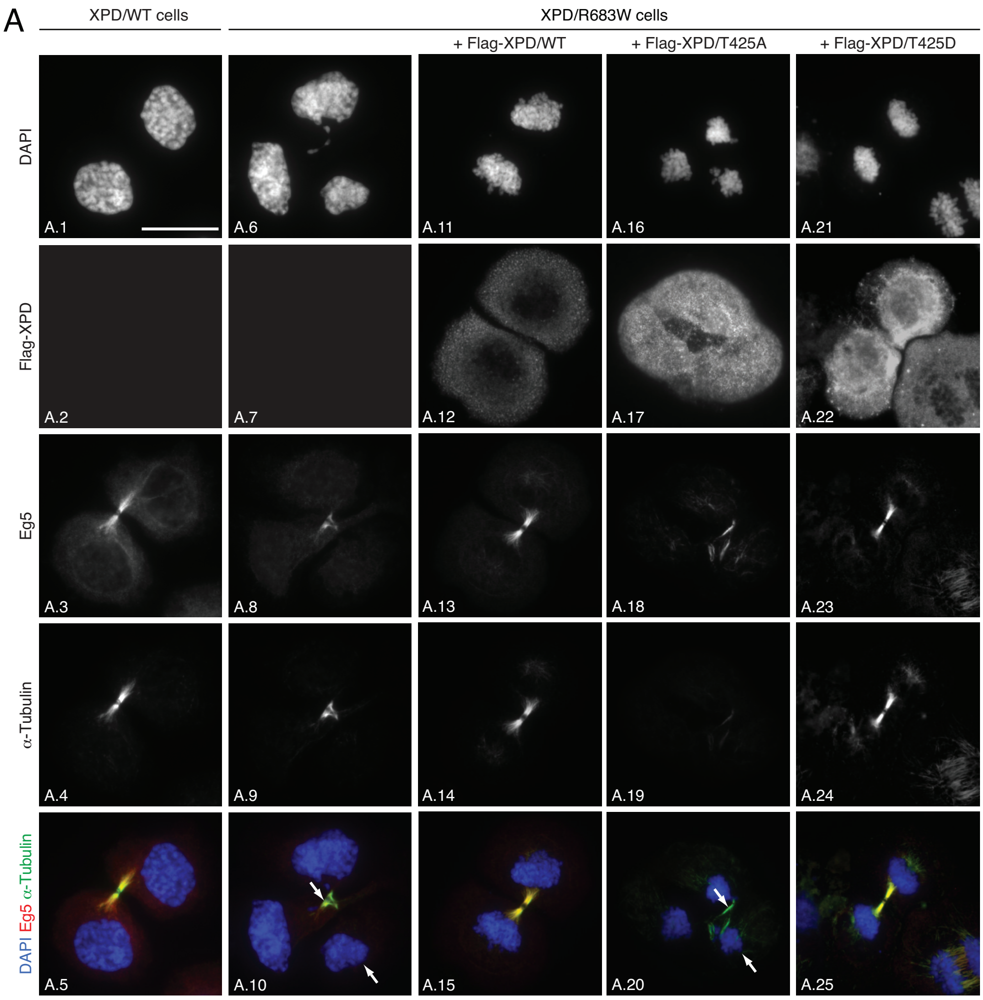
A
XPD/WT cells
XPD/R683W cells
+ Flag-XPD/WT
+ Flag-XPD/T425A
+ Flag-XPD/T425D
DAPI
Flag-XPD
Eg5
α-Tubulin
DAPI Eg5 α-Tubulin
A.1
A.2
A.3
A.4
A.5
A.6
A.7
A.8
A.9
A.10
A.11
A.12
A.13
A.14
A.15
A.16
A.17
A.18
A.19
A.20
A.21
A.22
A.23
A.24
A.25

Fig. S4. Localization of XPD, Eg5 and α-Tubulin in anaphase

(**A**) Unmerged images obtained with antibodies targeting the Flag-Tag, Eg5 and α-Tubulin in XPD/WT and XPD/R683W cells overexpressing either Flag-XPD/WT, /T425A or /T425D in anaphase. Chromosomes were stained with DAPI. The arrows point to DNA bridges. Scale bar is 5μm.

Figure S5

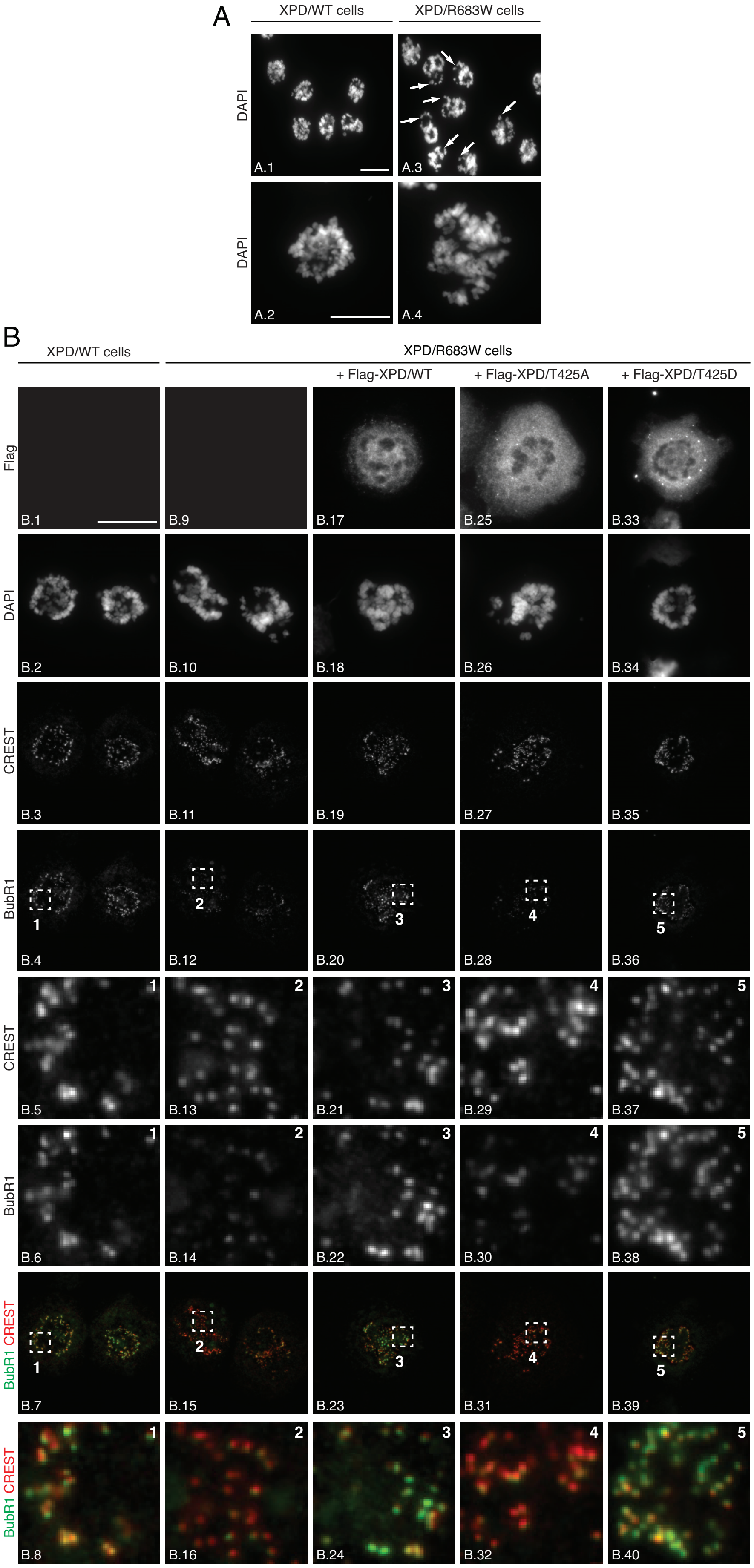

A
XPD/WT cells
XPD/R683W cells
DAPI
A.1
A.3
DAPI
A.2
A.4
B
XPD/WT cells
XPD/R683W cells
+ Flag-XPD/WT
+ Flag-XPD/T425A
+ Flag-XPD/T425D
Flag
DAPI
CREST
BubR1
CREST
BubR1
BubR1 CREST
BubR1 CREST
B.1
B.9
B.17
B.25
B.33
B.2
B.10
B.18
B.26
B.34
B.3
B.11
B.19
B.27
B.35
B.4
B.12
B.20
B.28
B.36
B.5
B.13
B.21
B.29
B.37
B.6
B.14
B.22
B.30
B.38
B.7
B.15
B.23
B.31
B.39
B.8
B.16
B.24
B.32
B.40

Fig. S5. CREST and BubR1 in XPD/WT and XPD/R683W cells in prometaphase.
(**A**) Wild-type and XPD mutated cells were synchronized in prometaphase with Taxol (16h, 1µM). Chromosomes were stained with DAPI. The arrows point to XPD/R683W anaphase-like cells that escaped prometaphase arrest in the presence of chromosome segregation errors. Scale bar is 5µm.
(**B**) Unmerged images for Flag-Tag, CREST and BubR1, from which merges presented Fig. 6E were made.

Figure S6

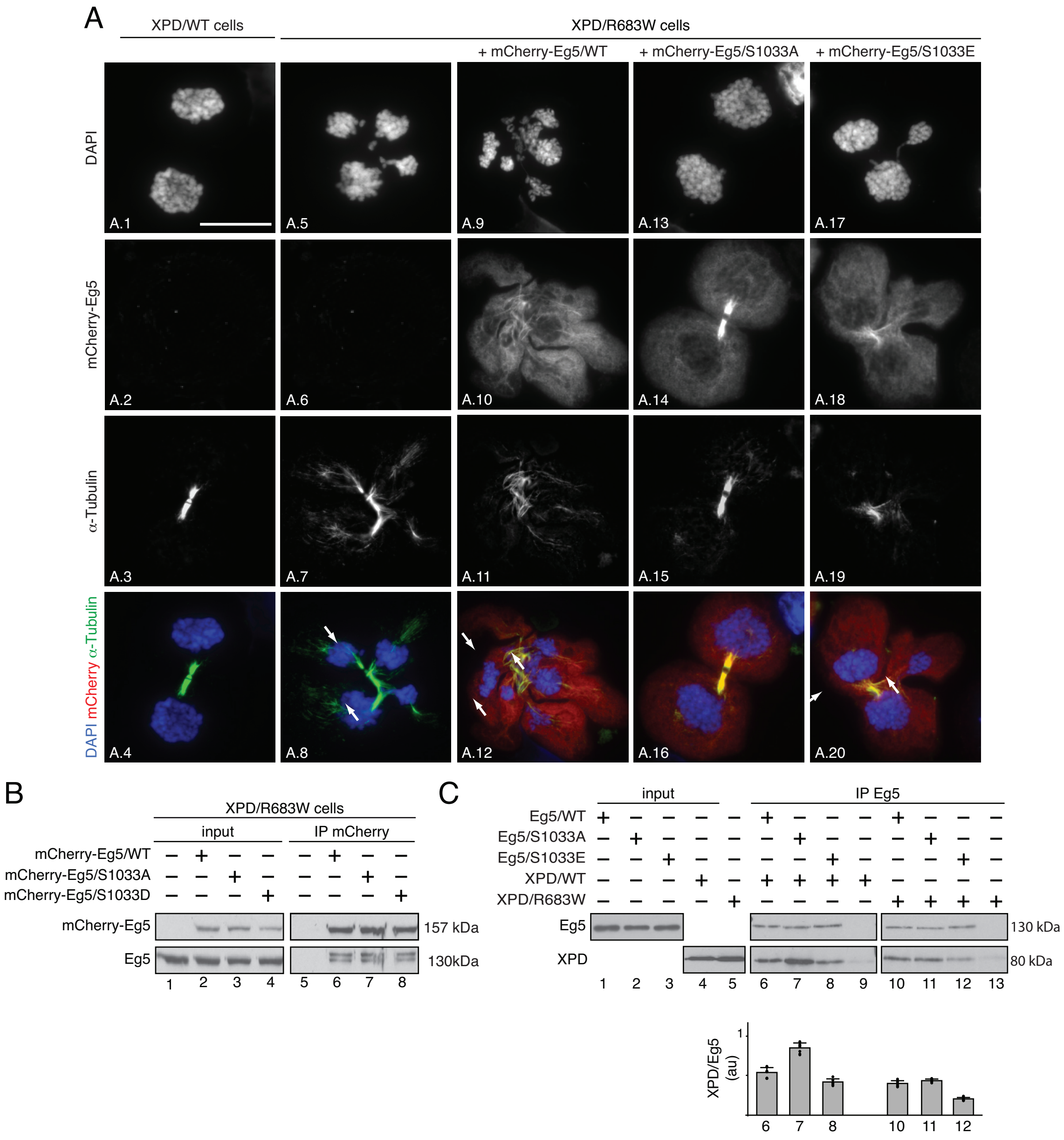

A
XPD/WT cells
XPD/R683W cells
+ mCherry-Eg5/WT
+ mCherry-Eg5/S1033A
+ mCherry-Eg5/S1033E
DAPI
mCherry-Eg5
α-Tubulin
DAPI mCherry α-Tubulin
A.1
A.2
A.3
A.4
A.5
A.6
A.7
A.8
A.9
A.10
A.11
A.12
A.13
A.14
A.15
A.16
A.17
A.18
A.19
A.20
B
XPD/R683W cells
input
IP mCherry
mCherry-Eg5/WT
mCherry-Eg5/S1033A
mCherry-Eg5/S1033D
mCherry-Eg5
Eg5
157 kDa
130kDa
1 2 3 4 5 6 7 8
C
input
IP Eg5
Eg5/WT
Eg5/S1033A
Eg5/S1033E
XPD/WT
XPD/R683W
Eg5
XPD
130 kDa
80 kDa
1 2 3 4 5 6 7 8 9 10 11 12 13
XPD/Eg5 (au)
1
6 7 8 10 11 12

Fig. S6. Localization in XPD/R683W cells of Eg5/WT, /S1033A and /S1033E in telophase

(**A**) Images obtained from XPD/WT and XPD/R683W cells overexpressing either the mCherry-Eg5/WT, /S1033A or /S1033E in telophase. Immunofluorescence analyses were performed with antibodies targeting the mCherry Tag and the mitotic spindle marker α-Tubulin. Chromosomes were stained with DAPI. Arrows point to DNA bridges. Scale bar is 5μm.

(**B**) Whole cell extracts were isolated from XPD/R683W cells overexpressing (when indicated, +) either mCherry-Eg5/WT, /S1033A or /S1033D. After immunoprecipitation with anti-mCherry antibody, the co-immunoprecipitated proteins were resolved by SDS-PAGE and blotted with anti-Eg5.

(**C**) When indicated (+) immunoprecipitated Eg5/WT, Eg5/S1033A or Eg5/S1033E was incubated with purified either XPD/WT or XPD/R683W. After washes, the co-immunoprecipitated proteins were resolved by SDS-PAGE and blotted with anti-Eg5 and XPD. Graph shows the ratio XPD/Eg5 (n=3, means ± s.d.) in arbitrary units (au).

Figure S7

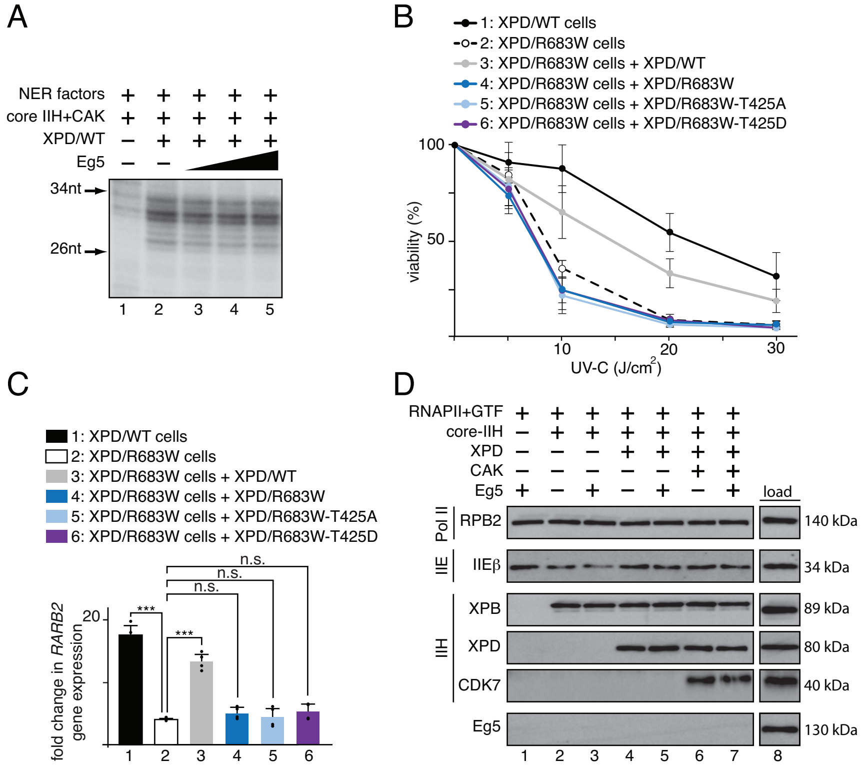
A
NER factors
core IIH+CAK
XPD/WT
Eg5
34nt
26nt
1 2 3 4 5
B
1: XPD/WT cells
2: XPD/R683W cells
3: XPD/R683W cells + XPD/WT
4: XPD/R683W cells + XPD/R683W
5: XPD/R683W cells + XPD/R683W-T425A
6: XPD/R683W cells + XPD/R683W-T425D
viability (%)
100
50
10 20 30
UV-C (J/cm2)
C
1: XPD/WT cells
2: XPD/R683W cells
3: XPD/R683W cells + XPD/WT
4: XPD/R683W cells + XPD/R683W
5: XPD/R683W cells + XPD/R683W-T425A
6: XPD/R683W cells + XPD/R683W-T425D
n.s.
n.s.
n.s.
***
***
fold change in RARB2 gene expression
20
1 2 3 4 5 6
D
RNAPII+GTF
core-IIH
XPD
CAK
Eg5
load
Pol II
RPB2
140 kDa
IIE
IIEβ
34 kDa
IIH
XPB
89 kDa
XPD
80 kDa
CDK7
40 kDa
Eg5
130 kDa
1 2 3 4 5 6 7 8

Fig. S7. Incidence of Eg5 and XPD/R683W in DNA repair and transcription.
(**A**) Increasing amounts of Eg5 were added to an incision/excision assay using purified recombinant NER factors (XPC, XPA, RPA, XPF/ERCC1 and XPG), the core-IIH, the CAK and XPD/WT. The reaction was analyzed by electrophoresis followed by autoradiography.
(**B**) Wild-type (XPD/WT) and XPD/R683W cells overexpressing either XPD/WT, XPD/R683W, XPD/R683W-T425A or XPD/R683W-T425D were treated with increasing UV-C doses and cells survival was determined 48h later. Data were normalized to the unexposed cells (as value of 100%). The results are the mean of 2 independent experiments performed in triplicates ± s.d. Significant statistical difference between XPD/R683W + XPD/WT cells and XPD/R683W, XPD/R683W + XPD/R683W, XPD/R683W + XPD/R683W-T425A or XPD/R683W + XPD/R683W-T425D at 10, 20 and 30J/cm^2 ($p<0.0001$, Student's t test).
(**C**) XPD/WT and XPD/R683W cells overexpressing either XPD/WT, XPD/R683W, XPD/R683W-T425A or XPD/R683W-T425D were treated 8h with t-RA (5µM) and relative *RARβ2* gene expression has been measured by RT-PCR. The mRNA levels were normalized to the GAPDH RNA amount. The *RAR*β*2* mRNA expression is presented as n-fold induction relative to non-treated cells. The results represent the mean of two independent experiments performed in triplicates. Bars 1-3 correspond to the values presented Fig. 8G.
(**D**) Biotinylated AdMLP bound to streptavidin magnetic beads was incubated with RNAPII, TFIIA,-B,-D (TBP),-E, and -F, in the presence of core-IIH, CAK, XPD and Eg5 as indicated at the top of the panel. After washes, the sequential binding of different factors (RPB2, TFIIEβ, XPB, XPD, CDK7, Eg5) in the PIC formation, was evaluated by immunoblotting.

Key Resources Table

REAGENTS and RESOURCES	SOURCE	IDENTIFIER
**Antibodies**		
mouse monoclonal anti-rabbit light chain-HRP	Jackson Immunoresearch	211-032-171
goat anti-mouse kappa-HRP	Southern Biotech	1050-05
rabbit polyclonal anti-Aurora B (immunoblotting)	Abcam	ab2254; AB_302923
rabbit polyclonal anti BubR1 (immunoblotting)	Invitrogen	720297; AB_2610165
mouse monoclonal anti CDK7 (immunoblotting)	IGBMC Antibody Facility	clone 2F8
human polyclonal anti CREST (immunostaining)	Antibodies Inc.	15-234; AB_2687472
rabbit polyclonal anti CCNB1 (immunoblotting)	GeneTex	GTX100911; AB_1949886
mouse monoclonal anti Cyclin H (immunoblotting)	IGBMC Antibody Facility	clone 2D4
rabbit polyclonal anti-Eg5 (immunoblotting)	Abcam	ab72413; AB_1268734
rabbit polyclonal anti-Eg5 (immunoblotting)	Abcam	ab61199; AB_941397
mouse monoclonal anti-Eg5 (immunoprecipitation)	Abcam	ab51976; AB_941398
mouse monoclonal anti-Eg5 (immunostaining)	Abcam	ab51976; AB_941398
rabbit polyclonal anti-Eg5 PhosphoT926	Abcam	ab61104; AB_942236
mouse monoclonal anti-Flag tag	Sigma-Aldrich	F1804; AB_262044
rabbit polyclonal anti-Flag tag	Sigma-Aldrich	F7425; AB_439687
rabbit polyclonal anti-GAPDH (immunoblotting)	Sigma-Aldrich	G9545; AB_796208
mouse monoclonal anti-GFP (immunoblotting)	Abcam	ab3277; AB_308705
rabbit polyclonal anti H3-pS10 (immunoblotting)	Cell Signaling	9701; AB_331535
normal mouse IgG (immunoprecipitation)	Santa Cruz	Sc-2025; AB_737182
normal rabbit IgG (immunoprecipitation)	Cell Signaling	2729S; AB_1031062
rat monoclonal anti mCherry (immunoprecipitation)	Fisher Scientific SAS	M11217; AB_2536611
rabbit monoclonal MMS19 (immunoblotting)	Cell Signaling	#90637; D5J8J
mouse monoclonal MMS19 (immunostaining)	IGBMC Antibody Facility	3MM3H10
mouse monoclonal anti NEK6 (immunostaining)	GeneTex	GTX84058; AB_10727078
rabbit monoclonal anti NEK6 (immunoblotting)	Abcam	ab109177; AB_10863726
rabbit monoclonal anti-phosphothreonine	Sigma-Aldrich	MABS499; clone RM102
rabbit monoclonal anti-PLK1 (immunoblotting)	Cell Signaling	4513S; AB_2167409
chicken polyclonal anti-α Tubulin (immunostaining)	Abcam	ab89984; AB_10672056
mouse monoclonal anti-α Tubulin (immunostaining)	Sigma-Aldrich	T9026; AB_477593
mouse monoclonal anti XPB (immunoblotting)	IGBMC Antibody Facility	clone 1B3
mouse monoclonal anti-XPD (immunoprecipitation)	Abcam	ab54676 ; AB_946174
rabbit monoclonal anti-XPD (D3Z6I) (immunoblotting)	Cell Signaling	11963; AB_2797781
rabbit polyclonal anti-XPD (immunoblotting)	Abcam	ab111596 ; AB_10863985
rabbit polyclonal anti-XPD [N2C2] (immunostaining)	GeneTex	GTX105357 ; AB_10616680
mouse monoclonal anti-XPG (immunoprecipitation)	IGBMC Antibody Facility	clone 1XPG1B5
rabbit polyclonal anti-XPG (immunostaining)	IGBMC Antibody Facility	3328
**Chemicals and Commercial Assays**		
Anti-Flag M2 Affinity Gel	Sigma-Aldrich	Cat#A2220
ATP	GE Healthcare Europe	27-2056-61
CDK1/CCNB1 (recombinant)	Abcam	ab104618
Chymotrypsin	Promega	V1061
CTP	Life Technologies SAS	R0451
DAPI (4′,6-Diamidino-2-phenylindole dihydrochloride)	Sigma-Aldrich	D8417
Dynabeads M-280 Streptavidin	Invitrogen	Cat#11206D

Dynabeads Protein G	Invitrogen	Cat#10004D
Monastrol	Sigma-Aldrich	M8515
Mowiol	Calbiochem	475904
NEK6 (recombinant)	Sigma-Aldrich	N4662
Nocodazole	Sigma-Aldrich	M1404
Paraformaldehyde 16%	Thermo Fisher	Cat#50-980-487
Prolong gold antifade mountant with DAPI	Fisher Scientific	P36935
TALON metal affinity resin	Clonetech	Cat#635501
Taxol (Paclitaxel)	Sigma-Aldrich	T7191
Thymidine	Sigma-Aldrich	T1895
Trypsin	Promega	V5111
X-treme GENE9	Roche Diagnostics	6365809001
**Cell Lines**		
HeLa (control cell line for HD2)	IGBMC Cell Culture Facility	
HD2 (bearing XPD/R683W)	IGBMC Cell Culture Facility	(*29*)
**Recombinant DNA**		
AdMLP DNA		(*55*)
mCherry-Kinesin11-N-18	Michael Davidson's lab	Addgene plasmid # 55067
pAK309		(*55*)
p-Flag Eg5/WT, /T926A, /T926D, /S1033A, /S1033E, /T926A-S1033A, /T926D-S1033E	this paper	N/A
p-mCherry Eg5/S1033A, /S1033E	this paper	N/A
pE-GFP Eg5 and pE-GFP XPD	this paper	N/A
pSK278-Flag MMS19/WT	this paper	N/A
pSK278-Flag XPD/WT, /R112H, R/683W /R722W,		(*41*)
pSK278-Flag XPD/T425A, and /T425D	this paper	N/A
**Primers**		
RARβ2	CCAGCAAGCCTCACATGTTTCCAA	TACACGCTCTGCACCTTTAGCACT
GAPDH	ACAACTTTGGTATCGTGGAAGG	GCCATCACGCCACAGTTTC
**Software**		
ImageJ 2.1.0	NIH	https://imagej.nih.gov/ij/
Prism 9.1.0	Graphpad	
Proteome Discoverer 2.4 software	Thermo Fisher Scientific	
**Other**		
Amersham Imager 600	GE Healthcare LifeSciences	29-0834-61
Cytospin 4 centrifuge	Thermoscientific	TH-CYTO4
Rotary Mixer	LABINCO	LD76
Thermomixer C	Eppendorf	035963

Caption for the Source Data File

This supplementary data file in Microsoft Excel format contains all the raw data and uncropped versions of any gels and blots presented in the figures.
